# OpcA and PorB are novel bactericidal antigens of the 4CMenB vaccine in mice and humans

**DOI:** 10.1038/s41541-023-00651-9

**Published:** 2023-04-12

**Authors:** Viola Viviani, Adele Fantoni, Sara Tomei, Sara Marchi, Enrico Luzzi, Margherita Bodini, Alessandro Muzzi, Marzia M. Giuliani, Domenico Maione, Jeremy P. Derrick, Isabel Delany, Mariagrazia Pizza, Alessia Biolchi, Erika Bartolini

**Affiliations:** 1 GSK, Siena, Italy; 2 Department of Pharmacy and Biotechnology, University of Bologna, Bologna, Italy; 3 Lydia Becker Institute of Immunology and Inflammation, School of Biological Sciences, Faculty of Biology, Medicine and Health, Manchester Academic Health Science Centre, University of Manchester, Manchester, M13 9PL UK

**Keywords:** Vaccines, Vaccines

## Abstract

The ability of *Neisseria meningitidis* Outer Membrane Vesicles (OMV) to induce protective responses in humans is well established and mainly attributed to Porin A (PorA). However, the contribution of additional protein antigens to protection remains to be elucidated. In this study we dissected the immunogenicity of antigens originating from the OMV component of the 4CMenB vaccine in mice and humans. We collected functional data on a panel of strains for which bactericidal responses to 4CMenB in infants was attributable to the OMV component and evaluated the role of 30 OMV-specific protein antigens in cross-coverage. By using tailor-made protein microarrays, the immunosignature of OMV antigens was determined. Three of these proteins, OpcA, NspA, and PorB, triggered mouse antibodies that were bactericidal against several *N. meningitidis* strains. Finally, by genetic deletion and/or serum depletion studies, we demonstrated the ability of OpcA and PorB to induce functional immune responses in infant sera after vaccination. In conclusion, while confirming the role of PorA in eliciting protective immunity, we identified two OMV antigens playing a key role in protection of infants vaccinated with the 4CMenB vaccine against different *N. meningitidis* serogroup B strains.

## Introduction

*Neisseria meningitidis* is an obligate human pathogen and is the major etiological agent of bacterial meningitis and sepsis worldwide. The bacterium inhabits asymptomatically the upper respiratory tract with a prevalence of 10–35% of healthy young adults in the global population^1^. Occasionally, it can progress into invasive meningococcal disease (IMD), which has the highest incidence in infants and adolescent^2^. Vaccination remains the only efficient approach to reduce disease burden and to limit long-term sequelae. Of the 12 serogroups of *N. meningitidis* present globally, six account for most disease cases worldwide and efficacious capsular polysaccharides vaccines have been licensed against four of them: A, C, W, and Y^3^. In contrast, the homology of the serogroup B capsule with human sialic acids resulted in its poor immunogenicity and also raised concerns of possible autoimmunity^4^. As an alternative approach, meningococcal Outer Membrane Vesicles (OMV) have been used to develop different tailor-made vaccines like *VA-MENGOCOC-BC*, *MenBvac*, and *MeNZB*, which successfully tackled clonal epidemics in Cuba, Norway and New Zealand, respectively^5–10^. Although the OMV challenges the human immune system with a composite mixture of antigens^11–17^, the protection provided by OMV-based vaccines has been primarily attributed to the immunodominant Porin A (PorA). This protein, characterized by tremendous antigenic variability and little cross-reactivity, is arranged in a conserved β-barrel structure spanning the outer membrane, with eight surface-protruding loops. Among them, loop 1 and loop 4, having the greatest antigenic diversity, are labeled as variable regions 1 and 2 (VR1 and VR2), respectively, and are largely responsible for PorA serosubtype-specific immune responses^18^. As a result, vaccines containing OMV provided poor coverage of heterologous strains expressing diverse PorA variants, especially in infants and young children^8^. Therefore, two protein-based recombinant vaccines have been developed and licensed against *N. meningitidis* serogroup B (MenB). rLP2086 (*Trumenba*) is a bivalent vaccine that contains two lipidated variants of the factor H binding protein (fHbp variant 1.55 and 3.45)^19^. 4CMenB (*Bexsero*) is a multicomponent formulation composed of three main recombinant antigens (rMenB): fHbp (variant 1.1) fused to the genome-derived *Neisseria* antigen (GNA) 2091, *Neisseria* Heparin Binding Antigen (NHBA, peptide 2) fused to GNA1030 and *Neisseria* adhesin A (NadA, variant 3). Besides these three proteins, the vaccine includes detergent-extracted OMV from the New Zealand epidemic strain (NZ98/254) harboring PorA as major antigen (P1.7-2,4)^20^. Apart from the extraction of loosely associated membrane lipoproteins and most of the lipooligosaccharide (LOS), the vesicles resemble the surface of the parental NZ98/254 strain and encompass the same array of outer membrane proteins (OMPs). According to SDS-PAGE analysis, five major classes of OMPs were identified in OMV^21^. However, recent proteomic-based approaches have revealed a complex OMV proteome which comprise up to 460 different proteins, mostly represented by OMPs, but also includes proteins predicted to be periplasmic and cytoplasmic^22–26^. The Porin B (PorB) represents the most abundant OMP present in the OMV of 4CMenB. It can be classified in two distinct and mutually exclusive classes designated as PorB2 and PorB3^27^; the latter representing the variant of the 4CMenB vaccine.

The low incidence of IMD has hampered accurate estimates of meningococcal vaccine efficacy. Consequently, the serum bactericidal assay (SBA) in the presence of exogenous human complement (hSBA) has been adopted as the “gold standard” laboratory-based surrogate of protection^28^. During clinical development, hSBA titers measured against four antigen-specific reference strains, one for each major vaccine antigens, has been routinely used to assess 4CMenB immunogenicity^29^. Therefore, although OMV contain multiple OMPs, their immunogenicity has been exclusively inferred based on anti-PorA antibodies testing NZ98/254 reference strain, underestimating the potential protective role played by additional OMV antigens. Nevertheless, encouraging 4CMenB early phase II studies conducted in infants revealed that the addition of OMV on top of rMenB was able to confer a broader protection when tested on a panel of seven MenB strains, some of which contained non-homologous 4CMenB antigens^30–32^. Several non-exclusive explanations have been proposed to support the observed protection: (1) synergy among antibodies targeting rMenB and/or OMV protein antigens; (2) an intrinsic immunostimulatory effect of OMV and its components; or (3) the role of antigens within the OMV component that might provide protection, in addition to PorA. While data supporting synergies occurring among rMenB-elicited antibodies targeting different proteins/epitopes and studies about neisserial OMV proteins as intrinsic adjuvants have been previously documented^33–38^, the immunological role played by antigens harbored by the OMV component of 4CMenB remain unclear, both at preclinical and clinical levels so far. A previous study demonstrated the feasibility of the use of an antigen microarray to identify IgG antibodies from adult human or mouse sera reactive against meningococcal OMPs^11^. The vaccine used in this study was an OMV preparation from a different *N. meningitidis* strain (H44/76), without the addition of the rMenB components. Here, we have adapted, extended, and complemented this approach to describe a functional analysis of the antibody response elicited by the OMV component of the 4CMenB vaccine in mice and in human vaccinees.

## Results

### The OMV component of 4CMenB induced bactericidal antibodies against a panel of OMV-specific strains

To assess the specific contribution of non-PorA OMV protein antigens from the overall immunological responses induced by the vaccine, we chose a panel of OMV-specific strains for which the cross-reactivity was not expected to be mediated by the recombinant vaccine components alone. In particular, for fHbp antigen limited protection against strains that express distantly related variants have been observed^39^. Differently, due to NHBA low expression levels, NHBA-elicited antibodies are able to mediate broader coverage when acting in concert with antibodies to other antigens, including OMV components, not necessarily bactericidal on their own^33,38^. For this reason, we selected and performed whole-genome sequencing of a panel of MenB strains mismatched for the individual antigens in the vaccine based on: (1) expressing fHbp variants dissimilar to the 4CMenB variant 1.1; (2) carrying NHBA peptide different from peptide 2; (3) not harboring the *nadA* gene or expressing a NadA variant other than variant 3; and (4) being mismatched for the vaccine PorA P1.7-2,4 variant. In Table 1 are listed the selected 12 strains which met the aforementioned criteria together with their associated metadata, vaccine antigen genotype and reactivity to the LOS homologous to that of the vaccine strain (NZ98/254—immunotype L3,7,9). In contrast, MC58, M10837 and NZ98/254 express the fHbp, NHBA, and PorA variants homologous to those within the vaccine, respectively.

**Table 1:** Characteristics of serogroup B meningococcal strains used as target strains in SBA.

Strain	Serogroup	Year of isolation	Country	Clonal complex	Sequence type	fHbp	*nadA*	NHBA	PorA VR1	PorA VR2	mAb L3,7,9^a^
NZ98/254	B	1998	NZL	41/44	42	1.14	Absent	2^b^	7-2^b^	4^b^	+
M10837	B	2003	USA	41/44	409	2.19	Absent	2^b^	18-1	34	−
LNP24651	B	2008	FRA	32	32	2.21	Yes (1)	47	7	16-26	+
M08389	B	2001	USA	162	162	2.21	Absent	20	22	14	+
M08117	B	2001	USA	41/44	437	2.19	Absent	29	7–4	1	+
MC58	B	1985	UK	32	74	1.1^b^	Yes (1)	3	7	16-2	+
M09662	B	2002	USA	60	60	1.13	Absent	191	21	16	+
M14569	B	2005	USA	35	35	2.16	Absent	21	22-1	14	+
M09929	B	2002	USA	35	3592	2.16	Absent	19	12-1	16	+
M07576	B	2000	USA	35	35	2.16	Absent	21	22-1	14	−
M12898	B	2004	USA	35	35	2.16	Absent	143	5-1	2–2	+
M07 241084	B	2007	UK	41/44	1097	2.302	Absent	31	19	15	+

*NZL* New Zealand, *USA* United States of America, *FRA* France, *UK* United Kingdom, *fHbp* Factor H binding protein, *nadA gene* *Neisseria* adhesin A, *NHBA* *Neisseria* heparin binding antigen, *PorA* Porin A, *VR* variable region.

The main characteristics of the isolates and the vaccine antigen genotype profile are listed.

^a^Recognition of the immunotype-specific mAb L3,7,9 is assessed via western blot on total cell extracts (Supplementary Fig. 1).

^b^Indicates when the isolate expresses variants of fHbp, NHBA and PorA homologous to those contained in the 4CMenB vaccine (fHbp variant 1.1, NadA variant 3, NHBA peptide 2, and PorA P1.7-2,4 serosubtype).

**Fig. 1:**
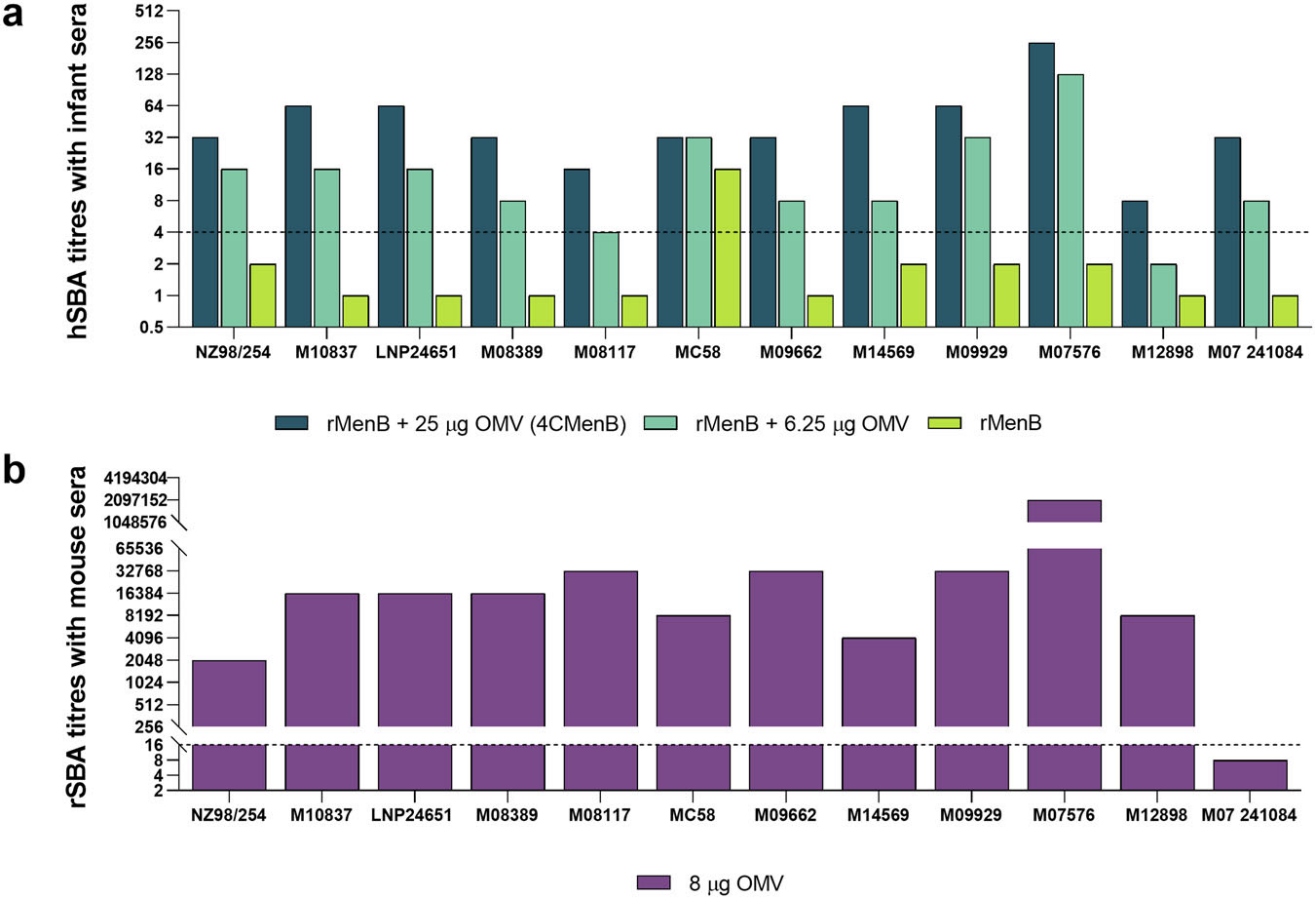
The OMV component of 4CMenB mediated bactericidal responses on vaccine heterologous strains. **a** Infant sera were collected and pooled (25 subjects each group) after the fourth vaccination dose and they were assayed in SBA with human complement. Solid bars indicate hSBA titers obtained against the 12 MenB strains using post-immunization rMenB + OMV (dark green), rMenB + 1/4 OMV (light green), rMenB (yellow-green) antisera. Bactericidal titers of preimmune sera were <2 for all groups against all tested strains. Titers ≥4 (dashed line) are considered protective. **b** rSBA titers were obtained using anti-OMV mouse sera against the panel of 12 MenB strains under investigation. Eleven groups of eight mice each deriving from distinct immunization schemes were pooled (*n* = 88) and used in the analysis. Serum bactericidal titers, indicating the dilution of the pooled mouse sera at which 50% of killing is reached, were determined using rabbit complement as source of complement. Titers ≥16 (dashed line) are considered positive as this dilution represents a fourfold increase respect to the lower limit of the assay corresponding to the first dilution tested (1:4).

The selected panel of 12 strains was used to investigate the functional immunogenicity of the OMV component of the 4CMenB vaccine. Standardized SBA in the presence of human complement was performed with pooled pre- and post-immunization sera deriving from three groups of 25 infants each that had received four doses of different vaccine formulations^32^. In particular, the three cohorts were immunized with: (1) rMenB alone (50 µg NadA, 50 µg NHBA-GNA1030, 50 µg GNA2091-fHbp); (2) rMenB + 1/4 OMV (rMenB + 6.25 µg OMV); or (3) 4CMenB containing the full OMV dose (rMenB + 25 µg OMV). Human SBA titers showed that all but one strain (MC58) were resistant to bacteriolysis when tested with the sera from the rMenB-vaccinated group (hSBA titers ≤2) (Fig. 1a). Killing of the MC58 strain was attributable to specific antibodies targeting the fHbp variant 1.1 expressed by the isolate. When the formulations containing OMV were tested in hSBA, strain coverage increased in a dose-dependent manner reaching 92% or 100% of strains killed with 6.25 µg or 25 µg of OMV, respectively (Fig. 1a). Owing to the unavailability of human sera derived from subjects vaccinated exclusively with OMV, we tested vesicle-driven protection with preclinical mouse anti-OMV antisera. Mice were immunized three times two weeks apart with 8 µg of OMV, and the resulting hyperimmune sera were tested in rabbit SBA (rSBA) against the same strains using rabbit complement. Positive bactericidal titers were obtained for 11 out of the 12 isolates, with titers ranging from 1/2048 to 1/2,097,152 (Fig. 1b), confirming the data with infant sera and demonstrating that the killing of these strains is conceivably mediated by OMV antigens other than PorA, working alone or in combination.

### OMV antigen selection, sequence conservation, and expression

To identify the specific OMV determinants mediating the functional cross-coverage, we investigated and prioritized a total of 30 OMV-specific antigens. They included 19 OMPs and 6 lipoproteins which were consistently identified as the most abundant in six different OMV production lots of 4CMenB (Table 2)^25^. Five additional antigens were also gathered from literature mining; in particular, the NspA protein was chosen as it represents the sixth most abundant antigen of the OMV-core proteome identified by a recent Nano-LC-MS/MS analysis, whereas PilW, ACP, OmpH and OmpH8 were previously reported as potential vaccine candidates^26,40–42^. PorA, considered the main immunogen of OMV, was included as a positive control. We then investigated the conservation of 28 out of the 30 antigens in the collection of 12 different strains, defining the percentage of sequence identity. MafA and PilE were excluded from the in silico analysis as these are known to be very heterogenic antigens in *Neisseria* spp. due to frequent intrachromosomal recombination^43,44^. The mean and the range of amino acid identity of the 28 proteins with respect to NZ98/254 are indicated in Table 2. At the amino acid level, 21 of the 28 proteins showed a sequence identity consistently >95%, 5 proteins >88% and PorB and NEIS0944 had the lowest identities (60.9% and 63.3%, respectively). In particular, the M09662 strain was the only one to carry the *porB2* allele, which is characterized by longer extracellular loops than *porB3*, the allele harbored by NZ98/254 and all the other strains. The low identity score detected for NEIS0944 antigen is ascribed to M08389 which had a shorter coding sequence due to a premature stop codon. Finally, our analysis revealed the absence of the *tbp1* in strain M14569. Overall, the high degree of sequence conservation of the antigens in the panel of isolates confirmed that the meningococcal strains selected were appropriate to investigate the role of OMV antigen in vaccine functionality.

**Table 2:** Main features of the 30 MenB proteins selected as potentially contributing to 4CMenB coverage.

Gene accession number PubMLST^a^ (MC58)^b^	Protein annotation	Protein abundance in OMV^c^	Aa sequence identity mean (%)^d^	Aa sequence identity range (%)^e^	*E. coli* GMMA SDS-PAGE /western blot^f^	Recombinant proteins SDS-PAGE^g^
Outer membrane proteins						
NEIS2020 (NMB2039)	Porin B; PorB	42.54	93.8	60.9–100	–/+	+
NEIS1364 (NMB1429)	Porin A; PorA	28.64	92.3	88.4–95.4	+/+	+
NEIS1428 (NMB1497)	TonB-dependent receptor	4.60	98.6	97.8–100	+/+	–
NEIS1783 (NMB0382)	Class 4 outer membrane protein; RmpM	3.08	99.4	98.8–100	–/+	+
NEIS0944 (NMB0964b)	TonB-dependent receptor	2.87	92.6	63.3–95.9	+/+	–
NEIS0408 (NMB1812)	PilQ protein	1.44	99.2	96.9–100	+/+	–
NEIS1963 (NMB1988)	Iron-regulated outer membrane protein; FrpB (FetA)	0.96	94.8	91.1–98	+/+	–
NEIS1690 (NMB0461)	Transferrin-binding protein 1; Tbp1 (TbpA)	0.92	96.3*	95.3–97*	–/–	–
NEIS0173 (NMB0182)	Outer membrane protein Omp85	0.87	99.4	98.9–100	+/+	–
NEIS2198 (NMB1053)	Class 5 outer membrane protein; OpcA	0.75	98.4	98.2–98.9	+/+	–
NEIS0073 (NMB0088)	Outer membrane protein P1; OmpP1	0.54	95.4	93.4–100	+/+	–
NEIS1468 (NMB1540)	Lactoferrin-binding protein A; LbpA	0.46	97	94.7–98.9	+/+	–
NEIS0275 (NMB0280)	Organic solvent tolerance protein; OstA	0.44	99.2	98–99.8	–/–	–
NEIS1632 (NMB1714)	Multidrug efflux pump channel protein; MtrE	0.29	99.5	98.8–100	+/+	+
NEIS0101 (NMB0109b)	Hypothetical protein	0.26	99.5	99.3–100	–/–	–
NEIS1271 (NMB1333)	Hypothetical protein	0.24	99.6	98.7–100	+/+	–
NEIS1487 (NMB1567)	Macrophage infectivity potentiator; MIP	0.23	99.7	97.8–100	+/+	+
NEIS1687 (NMB0464)	Phospholipase A1	0.09	99.6	99.2–100	–/+	–
NEIS0210 (NMB0018)	Pilin PilE	0.08	n/a	n/a	–/–	−
NEIS2075 (NMB2095)	Adhesin complex protein; ACP	n/a	99.9	99.2–100	+/+	+
NEIS1246 (NMB1309)	Fimbrial biogenesis and twitching motility protein, PilW	n/a	99.8	99.2–100	–/+	+
NEIS0612 (NMB0663)	Outer membrane protein, NspA	n/a	99.2	97.7–100	+/+	–
NEIS1462 (NMB1533)	Outer membrane protein H.8; OmpH8	n/a	99.1	95.8–100	+/+	+
NEIS0172 (NMB0181)	Outer membrane protein H; OmpH	n/a	99.9	99.4–100	+/+	–
Lipoproteins						
NEIS1066 (NMB1126/NMB1164)	Hypothetical protein	1.06	99.6	99.1–100	+/+	+
NEIS0596 (NMB0375/NMB0652)	MafA protein	0.18	n/a	n/a	+/+	–
NEIS0653 (NMB0703)	Competence lipoprotein; ComL	0.14	99.5	96.3–100	+/+	+
NEIS1634 (NMB1716)	Membrane fusion protein; MtrC	0.12	99.2	98.5–99.8	–/+	+
NEIS0196 (NMB0204)	Outer membrane protein assembly factor; BamE	0.10	94.7	89.7–95.2	+/+	+
NEIS1065 (NMB1125/NMB1163)	Hypothetical protein	0.06	99.9	99.2–100	+/+	+

*Aa* amino acid.

^a,b^Gene accession numbers are provided as NEIS, as encoded in PubMLST (https://pubmlst.org/neisseria/), and as NMB, as reported in NCBI for MC58 (https://www.ncbi.nlm.nih.gov/nuccore/NC_003112.2).

^c^Average of protein abundance calculated analyzing six OMV production lots of 4CMenB from ref. ^25^. Data not available from ref. ^25^ are described as n/a.

^d,e^Pairwise sequence alignments of the 30 OMV-specific proteins in the 11 MenB strains versus NZ98/254 were carried out, and the percentages of identity (mean and range) of each antigen are reported. n/a denotes the unavailability of nucleotide sequence. The asterisk (*) indicates that the data refer to 10 strains since the gene *tbp1* is absent in M14569.

^f^The expression of MenB proteins in *E. coli* GMMA is represented by a “+” or “–” if a band was or was not detected in the SDS-PAGE and western blot (α-FLAG antibodies) according to data described in Supplementary Fig. 2b, c, respectively.

^g^The availability of purified recombinant MenB protein is represented by a “+” according to data described in Supplementary Fig. 4. When the recombinant protein was not purified it is indicated by a “–”.

To allow a native folding of integral OMPs, *Escherichia coli* OMV derived from strains engineered to hypervesiculate, hereafter named GMMA (Generalized Modules for Membrane Antigens), were used as expression tool. The coding regions of the prioritized antigens were amplified from the vaccine strain and cloned in fusion with the *E. coli* OmpA leader sequence into an IPTG-inducible expression plasmid (Supplementary Fig. 2a). The NEIS0101 and PilE proteins were cloned instead with their natural secretory signal, and all the resulting constructs were used to transform the *E. coli* mutant strains BL21(DE3) Δ*tolR* or BL21(DE3) Δ*ompA*. Bacterial strains transformed with vector without any insert were used to produce empty GMMA as negative controls. The expression of MenB antigens in the resulting *E. coli* GMMA was verified by Coomassie blue-stained gels and western blots probed with anti-FLAG antibodies (Supplementary Fig. 2b, c, respectively). As shown by the SDS-PAGE, 21 of the 30 prioritized proteins were expressed at different levels in *E. coli* GMMA once fused to amino- or carboxy-terminal hexahistidine and FLAG tags. Meningococcal antigen expression in GMMA of five other proteins was further confirmed by immunoblots, for a total of 26 expressed antigens out of the 30 selected. We were unable to express PilE, Tbp1 and OstA in GMMA, despite the fact that the proteins were present in the corresponding whole-cell lysates. On the contrary, NEIS0101 lacked significant expression also in cell lysates (Supplementary Fig. 3). In parallel with expression trials of antigens in *E. coli* GMMA, we explored the possibility to obtain meningococcal proteins in the soluble form. Total and soluble fractions of lysates from the expression cultures were screened in western blot for protein expression and from them, proteins that resulted soluble were selected for purification. Conversely, PorA and PorB were expressed in the inclusion bodies, resolubilized and refolded by dilution into buffers containing detergents before purification. From the panel of the 30 MenB antigens 13 different recombinant proteins were successfully purified as shown in the SDS-PAGE of Supplementary Fig. 4. A detailed summary of the expression and purification results of meningococcal antigens is listed in Table 2.

### The most immunogenic OMV antigens were identified by protein-microarray analysis

**Fig. 2:**
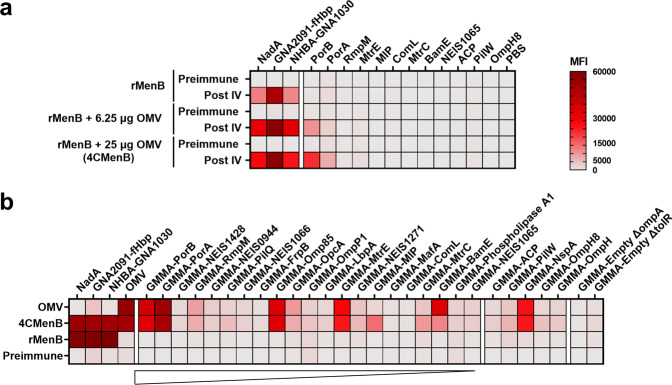
The immunosignature of human and mouse antisera revealed the immunodominant OMPs of OMV. **a** Infant sera collected before and after the fourth dose of different vaccines were assayed as pool on a microarray containing exclusively recombinant proteins. **b** Pre- and post-immunization mouse sera were pooled (preimmune pool *n* = 80, rMenB pool *n* = 10, 4CMenB pool *n* = 54, OMV pool *n* = 88) and probed onto a microarray spotted with engineered *E. coli* GMMA. Samples are ranked according to the relative abundance of antigens in OMV^25^ as shown by the triangle. Each tinted block represents the averaged reactivity of the replicated spots for each serum screened, and results are expressed as MFI values. Signals were considered positive when their MFI were greater than 5000, corresponding to the MFI of control protein spots after detection with fluorescent-labeled secondary antibodies, plus ten times the standard deviation. Three arbitrary MFI thresholds were also assigned for low (5000 ≤ MFI < 15,000), medium (15,000 ≤ MFI < 30,000 MFI) and high (MFI ≥ 30,000) reactivities. Color scale of signal intensity is reported on the right of the heatmap.

To gain insights on the humoral response induced by the different components of the serogroup B meningococcal vaccine, a protein array tailored to the OMV component of 4CMenB was generated and used to characterize the polyclonal antibody reactivity. Two separate protein microarrays were printed: one containing all the recombinant OMV antigens available and the other with the engineered *E. coli* GMMA, the two GMMA empty negative controls and the vaccine OMV. As a positive control, the rMenB components of 4CMenB were spotted onto nitrocellulose-coated glass slides of both microarrays. After the validation step, immunoprofiling studies were carried out with pooled human and animal sera immunized with different vaccine formulations. Preimmune sera were included in the analysis to evaluate the background signal. Serum reactivity was assessed by detecting total IgG bound to each spot using fluorescently labeled secondary antibodies and measuring the mean fluorescence intensity (MFI) values of each protein. Hybridization results were summarized in the heatmaps shown in Fig. 2. Infant sera raised against 4CMenB showed high reactivity on *E. coli* GMMA and were positive for all GMMA-based proteins on the array, likely due to pre-existing immunity against *E. coli* related antigens. Human results on the GMMA array were thus considered unreliable. Nevertheless, the reactivity of human sera to recombinant proteins spotted on the protein array highlighted the presence of signals mainly against the rMenB component of 4CMenB and against the major OMV proteins PorA and PorB (Fig. 2a). With mouse sera, the immunological fingerprint depended on the different vaccine formulations used for the immunization studies (Fig. 2b). As expected, IgG antibodies raised by rMenB were exclusively directed against NadA, GNA2091-fHbp and NHBA-GNA1030. By contrast, following immunization both with 4CMenB and OMV, we found antibodies mostly reacting with PorA, PorB, RmpM, BamE, MtrE, NspA, OpcA, and Omp85. Interestingly, the immunosignature obtained with the 4CMenB vaccine was nearly identical to the one obtained with the single OMV component. Moreover, no clear correlation between immunogenicity and antigen amount contained in OMV was observed: BamE and MtrE present in OMV in very low quantity (0.1 and 0.29% of the total protein amount, Table 2)^25^, showed high IgG reactivity. In summary, a specific subset of OMV antigens able to induce a robust immune response both when administered alone and when included in the 4CMenB formulation was identified.

### A specific antibody response was induced by OMV antigens in mice

To investigate the ability of the non-PorA OMV antigens to induce functional antibodies, mice were immunized intraperitoneally three times at two weeks apart with each single recombinant protein or with each *E. coli* GMMA expressing heterologous antigens. As negative and positive controls, mice were immunized with the two Empty GMMA or with PorA-GMMA, respectively. Serum samples collected two weeks after the last immunization were initially characterized in western blots against OMV and whole-cell extracts from NZ98/254 and against crude lysates of M07576 and M09929. As shown in Supplementary Fig. 5a, sera raised against Empty GMMA did not induce antibodies able to recognize meningococcal proteins. On the contrary, 22 of the 26 antigens elicited antibodies that specifically recognize the native protein expressed by the isolates and/or present in OMV (Supplementary Fig. 5b–d). The lack of reactive bands at the expected molecular weight for FrpB, NEIS1428, Phospholipase A1 and NEIS1271 might be due to suboptimal expression levels of the meningococcal antigens in GMMA (Supplementary Fig. 5c). Intriguingly, all the 12 antigens used for immunization both as recombinant proteins and expressed in GMMA triggered the production of specific antibodies (Supplementary Fig. 5d). Furthermore, the availability of recombinant proteins allowed us to measure antigen-specific IgG titers in individual mouse serum by ELISA. The geometric mean titers shown in Supplementary Fig. 6 confirmed the induction of specific antibodies in all antisera assayed. In particular, 10 of the 13 recombinant GMMA showed IgG titers significantly higher with respect to the baseline recorded from the GMMA control antisera (Supplementary Fig. 6). Densitometric analysis of comparative western blots (Supplementary Fig. 7) performed on four antigens expressed at different levels in GMMA and used as representative expression profiles (ComL: high, MIP: medium, BamE: low, PilW: no expression, according to SDS-PAGE of Supplementary Fig. 2) indicated that the specific antigen approximately corresponds to 18%, 2.1%, 3.2%, and 0.27% of the total protein content, respectively. Overall western blots and ELISA data suggest that antigen-specific and cross-reactive antibodies were elicited in almost all sera.

### PorA, OpcA, PorB, and NspA induced bactericidal antibodies in mice

**Fig. 3:**
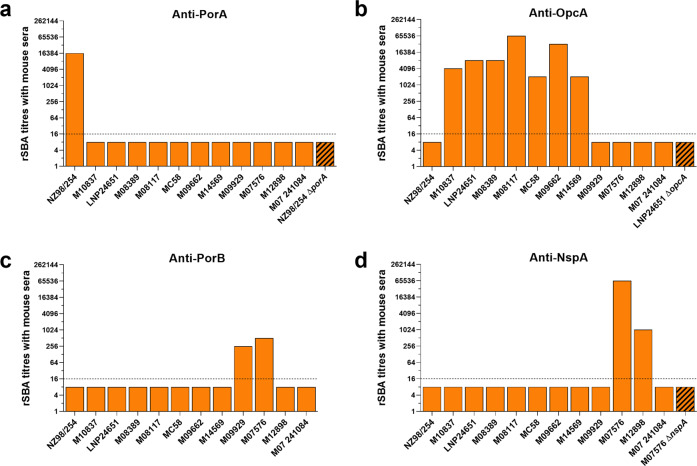
PorA, OpcA, PorB, and NspA antibodies showed strain-specific bactericidal killing. rSBA titers obtained using GMMA-PorA (**a**), GMMA-OpcA (**b**), rPorB (**c**), and GMMA-NspA (**d**) antisera are represented by histograms. Filled bars represent natural strains, while stripped bars indicate knockout mutants used as negative controls. rSBA titers ≥16 are considered positive (dashed lines). Sera used in all the analyses are pool of eight mice.

We then verified the ability of antibodies to induce complement-mediated killing of the OMV-specific strains. SBA analyses in the presence of rabbit complement were initially performed using sera raised against the eight antigens giving the highest reactivity in protein array when probed with OMV antisera. While no bactericidal activity was observed for Omp85, BamE, MtrE and RmpM antisera (rSBA titers <16), antisera against PorA, OpcA, PorB, and NspA showed high bactericidal activity (Fig. 3). As expected, anti-PorA sera induced bactericidal titers only against the homologous vaccine strain NZ98/254, but not against the 11 heterologous strains nor against the *porA*^-^ NZ98/254 isogenic knockout strain (Fig. 3a). Interestingly, OpcA, PorB and NspA antisera showed a strain-specific bactericidal pattern. In the case of OpcA, the antisera mediated the killing of seven out of 11 heterologous isolates with titers ranging from 2048 to 65,536 (Fig. 3b). Antibodies against PorB and NspA elicited bactericidal activity against two out of the 11 strains (M09929-M07576 and M07576-M12898, respectively), which were not killed by anti-OpcA antibodies (Fig. 3c, d, respectively). The isogenic knockout mutant strains (LNP24651 Δ*opcA* and M07576 Δ*nspA*) were not killed in SBA confirming the specificity of the functional response. In the case of PorB, we did not succeed in generating PorB-deficient M07576 and M09929 strains. As detailed in Supplementary Table 1, none of the antisera against other antigens was able to provide functional immunity against the NZ98/254, M07576, and M09929 strains. To conclude, we identified the OMV antigen responsible for the bactericidal activity for 11 of the 12 OMV-specific strains.

**Fig. 4:**
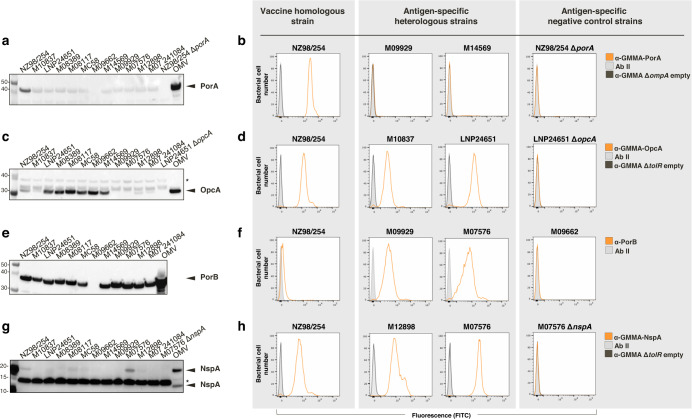
Evaluation of the expression levels and surface-localization of PorA, OpcA, PorB, and NspA in the panel of OMV-specific strains. OMV and meningococcal crude lysates, prepared from bacteria culture grown in SBA-like conditions, were resolved in SDS-PAGE prior to western blotting and probing with GMMA-PorA (**a**), GMMA-OpcA (**c**), rPorB (**e**), and GMMA-NspA (**g**) sera. Specific bands are indicated by arrows. Asterisk refers to the unspecific band used as loading control among strain lysates. The binding of polyclonal antisera raised by PorA (**b**), OpcA (**d**), PorB (**f**), and NspA (**h**) antigens was assessed by flow cytometry against four MenB strains (the vaccine strain NZ98/254, two representative heterologous strains for each antigen and the specific negative control strain). The whole panel of strains assayed in FACS surface staining is represented in Supplementary Fig. 8. Shaded gray profiles represent bacterial cells exclusively stained with secondary antibodies, while non-shaded dark gray profiles indicate sera raised against GMMA empty Δ*tolR* and Δ*ompA* (negative controls). Non-shaded orange profiles show the reaction with specific immune sera. Ab II secondary antibody. The uncropped and unprocessed scans are reported in Supplementary Fig. 9.

To further understand the strain-specific susceptibility to the killing obtained with the four different bactericidal sera, we investigated antigen expression levels and surface exposure together with serum cross-recognition in the complete panel of OMV-specific strains (Fig. 4 and Supplementary Fig. 8). The immunoblot of crude cell extracts revealed that the PorA antisera recognized the homologous antigen expressed by NZ98/254 or present in the OMV. On the contrary, we observed little or no recognition for the heterologous PorA alleles (Fig. 4a), in line with the different VR1 and VR2 epitopes harbored by the strains. In agreement, FACS analysis revealed that the PorA antisera have strong binding ability only on NZ98/254 (Fig. 4b and Supplementary Fig. 8a) and rSBA data confirmed the variant specificity of PorA antibodies. As shown in Fig. 4c, the amount of OpcA protein across the strains was variable and ranged from high (OpcA^++^: LNP24651, M08389, M08117, MC58, M09662, M14569), to intermediate (OpcA^+^: NZ98/254, M10837, M12898, M07 241084) to negative levels (OpcA^-^: M09929 and M07576 although carrying the *opcA* gene and LNP24651 Δ*opcA*). Similarly, flow cytometry analyses showed a differential surface exposure of OpcA among the tested strains (Fig. 4d and Supplementary Fig. 8b). Given the high percentage of amino acid sequence identity shared by OpcA in the bacterial collection (98.2–98.9% of amino acid identity, Table 2), different OpcA reactivities may imply different expression levels as detected in the western blots. Altogether, these results suggested that meningococcal isolates that express higher relative quantities of total protein are more susceptible to killing than strains expressing lower amounts. Western blot experiments performed using anti-PorB sera revealed comparable levels of PorB expression for 11 of the 12 MenB isolates which carry the same PorB3 vaccine allele (Fig. 4e). As expected, the M09662, the only strain expressing the PorB allele 2, was negative; this strain was also selected as negative control for FACS surface staining experiments. FACS highlighted a marked surface staining of the PorB protein to antibodies in the M09929 and M07576 isolates and an absence or limited surface staining in the other isolates, regardless of the similar expression levels showed in the immunoblot (Fig. 4f and Supplementary Fig. 8c). This may explain the PorB-dependent bacteriolysis observed for M09929 and M07576 in which the PorB target antigen is accessible on the meningococcal surface and the absence of killing for the NZ98/254 strain and likely for the others. Unlike the other antigens, immunoblots analysis of NspA did not provide straightforward results. Indeed, in the western blot with NspA antisera a protein of ∼18.5 kDa corresponding to the expected molecular weight of NspA was only detected in M07576 whole-cell lysates. A signal corresponding to ∼15 kDa present in all lysates was unspecific since present also in the M07576 *nspA* deletion mutant (Fig. 4g). Considering the very high amino acid sequence identity of NspA among our strains (97.7–100%, Table 2), we excluded differences in cross-recognition caused by polymorphisms of NspA protein. Indeed, FACS analysis showed surface exposure of NspA in most of the tested strains (Fig. 4h and Supplementary Fig. 8d). In conclusion, there is not a complete correlation between SBA results and expression levels or surface exposure of NspA protein which may explain the differential susceptibility to the killing observed among the isolates. Conversely, expression of OpcA and surface accessibility of PorB are clearly related to strain susceptibility to bactericidal killing.

### Specificity of the PorA, PorB, and OpcA bactericidal activity in human vaccinee sera

**Fig. 5:**
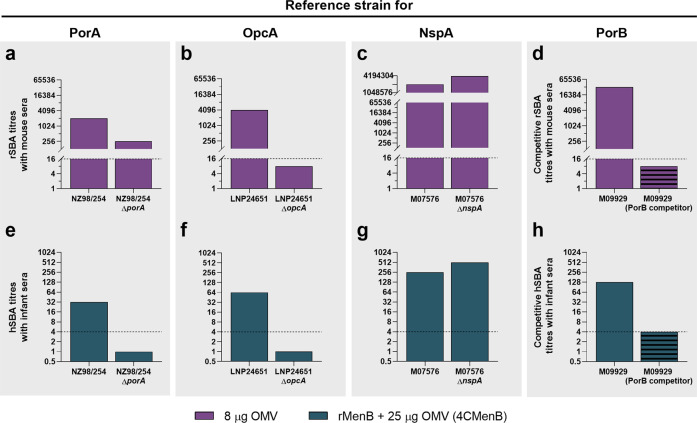
Anti-PorA, -OpcA, and -PorB functional antibodies were raised by the OMV component of the 4CMenB vaccine in humans. **a**–**c** rSBA titers obtained with pooled OMV mice sera against wild-type and knockout strains are represented by columns. Dashed lines represent the minimal threshold of functionality observed in the presence of baby rabbit complement. **d** The ability of PBS buffer alone or purified recombinant PorB protein to inhibit the killing of M09929 in the presence of OMV pooled sera was tested by competitive SBA and the resulting rSBA titers are illustrated as filled or striped histograms, respectively. Competitor is indicated in brackets. rSBA ≥16 are considered positive (dashed lines). **e**–**g** The ability of pooled (*n* = 25) infant immune sera collected from infants who received four doses of 4CMenB to kill wild-type and knockout *N. meningitidis* strains was tested in hSBA. Bactericidal titers are described as columns. hSBA ≥4 are considered protective (dashed line). **h** Competitive hSBA was performed on M09929 using PBS or PorB protein as a competitor (filled and striped bars, respectively).

To confirm the ability of PorA, PorB, OpcA, and NspA to elicit functional antibodies from the vaccine OMV, we performed SBA analysis. The assay was carried out on specific reference strains using mouse OMV antisera and rabbit complement (rSBA) or human 4CMenB vaccinee sera and human exogenous complement (hSBA), Fig. 5a–h, respectively. NZ98/254, LNP24651, and M07576 and their isogenic knockout strains were chosen to measure the specific contribution of PorA, OpcA, and NspA, respectively, while the specific contribution of PorB was evaluated through competitive SBA, due to the unavailability of the knockout mutants. As expected, rSBA titers between NZ98/254 and the corresponding *porA*^*−*^ mutant differed by threefold (2048 and 256, respectively), as shown in Fig. 5a. This result indicates that PorA is the main immunodominant antigen of the OMV and suggests that the residual killing ability on the NZ98/254 Δ*porA* strain might be due to bactericidal antibodies targeting minor non-PorA OMV antigens, confirming their role in inducing functional antibodies. In the case of OpcA the bactericidal activity against the LNP24651 strain (rSBA titer = 4096) was completely abolished on the *opcA* knockout mutant (Fig. 5b), suggesting that the killing of this strain was solely mediated by anti-OpcA antibodies. In contrast, NspA deletion in the M07576 strain had no effect on the killing ability of OMV antibodies, with very high bactericidal titers observed against both strains (M07576 and M07576 Δ*nspA*) (Fig. 5c). To measure the contribution of PorB-specific antibodies in the killing mediated by OMV-sera, competitive SBA was performed on the M09929 isolate by adding the PorB protein as competitor (Fig. 5d). The rSBA titer decreased from 32,768 against M09929 strain to 8 when sera were pre-treated with recombinant PorB. This result provided evidence for the specific immunogenic activity of PorB within OMV and identified this antigen as the major OMV component responsible for M09929 killing. Finally, having demonstrated the direct role of anti-PorA, -OpcA, and -PorB antibodies in OMV-mediated killing in mice, we confirmed their ability to induce protective responses in human subjects following 4CMenB vaccination. Interestingly, anti-PorA, anti-OpcA and anti-PorB antibodies induced in infants vaccinated with 4CMenB were able to mediate a specific killing of the NZ98/254, LNP24651, and M09929 strains, respectively (Fig. 5e, f, h). On the contrary, the contribution of anti-NspA antibodies induced in infants was not measurable (Fig. 5g). These results confirm the role played by PorB and OpcA in the induction of bactericidal antibodies in infants. Moreover, predicted protection against NZ98/254, M07576, and M09929 was dependent upon one of these antigens contained exclusively within OMV, supporting the pivotal role played by these vesicles in the 4CMenB multivalent formulation.

## Discussion

Preclinical and clinical studies performed after vaccine licensure have progressively deepened the knowledge on the three major recombinant antigens of 4CMenB. In contrast, little is known about the immunogenicity of the multiple antigens contained in the vaccine OMV, apart from the immunodominant nature of PorA. Indeed, after monovalent OMV vaccinations, PorA-specific immunoglobulins tend to dominate the antibody responses^8,18^. Nevertheless, the ability of 4CMenB to kill several vaccine-mismatched strains suggested that OMV proteins other than PorA contributed to the full extent of protection against different *N. meningitidis* strains^31,32,45^. In this study we identify PorB and OpcA as additional bactericidal 4CMenB antigens that contribute to the breadth of protection in humans. In contrast, while NspA was shown to induce antibodies following human infection in previous studies^46^, we were unable to show its direct contribution in bactericidal killing by human sera. According to our study, NspA antibodies raised in mice were instead able to mediate bactericidal killing. The role of the OMV component in the 4CMenB vaccine was determined by measuring OMV-specific bactericidal responses on an ad hoc panel of 12 genetically distinct MenB strains, selected as a subset of the 61 strains recently used to demonstrate extended coverage of 4CMenB^38^. By using meningococcal vaccine formulations with or without OMV components, we demonstrated that antibodies targeting OMV antigens are essential for the killing of the selected strains. In infants, the age group at greatest risk of contracting IMD, we observed that OMV-containing formulations elicited higher bactericidal titers than rMenB antigens alone against the tested strains, suggesting a more robust and a broader range of predicted protection. Breadth of protection is particularly significant in this infant cohort, which is likely naïve. As such, we expect the functional responses measured against the 12 OMV-indicator strains to be vaccine-induced. It has been hypothesized that pre-existing immunity to meningococci, acquired during childhood and youth by repeating colonization of neisserial commensal or pathogenic species, may be boosted by OMV vaccines^47^. In particular, while in naturally primed older age groups even small amounts of antigens present in OMV may be sufficient to boost protective responses, in naïve infants this is rather unlikely^48,49^. Despite mouse immune responses may not completely recapitulate those found in humans, we conducted immunogenicity studies in mice naïve for meningococci. Through these animal studies, we further corroborated the protection on the OMV-specific strain panel as anti-OMV mice sera were bactericidal against 11 of the 12 strains. Interestingly, the only strain resistant to killing with mouse OMV antisera was the M07 241084 isolate, which, in contrast, was susceptible to sera from 4CMenB vaccinees. Since this strain is mismatched to the fHbp variant present in 4CMenB and lacks the *nadA* gene, the killing could be due to synergistic mechanisms between OMV and the other components of 4CMenB as previously described^38^. Likewise, although the NZ98/254 and M10837 strains harbor the homologous NHBA vaccine peptide, our negative hSBA results with rMenB human sera are in line with previous observations that NHBA low expressor strains are killed mainly when anti-NHBA antibodies act synergistically with antibodies targeting other antigens^34,35,38,50^.

The complex nature of OMV poses several challenges for the deconvolution of antigen-specific contributions to the overall vaccine efficacy. Here, we narrowed the investigation to a prioritized list of 30 OMPs and lipoproteins according to their presence, abundance and cellular localization. In abundance, 21 of these proteins represent approximately 90% of the OMV content^25^. In order to overcome the issues for expression and folding of OMPs in their native conformation essential to maintain protective epitopes^51^, we expressed them in their membrane configuration in GMMA which have been shown to represent an efficient tool for the expression of bacterial antigens^52^. From the immunoprofiling of mice and human sera on an OMV-tailored microarray spotted with the individual proteins, we identified eight immunogenic antigens which interestingly did not correlate with their abundance in the OMV. In our screening of human sera, only the major porin proteins of OMV (PorA and PorB) were immunoreactive, while in mice, also RmpM, BamE, MtrE, NspA, OpcA, and Omp85 generate strong IgG responses. In a recent study, a similar immunological fingerprint was performed with sera from mice and adults administered with an OMV vaccine (*MenPF1*) on a microarray composed of 91 native and refolded OMPs^11^. The authors identified four integral OMPs (PorA, PorB, OpcA, and PilQ), two lipoproteins (BamC and GNA1162) and RmpM as the top responding antigens in humans, while only PorA, PorB, and PilQ were targeted by mouse immunoglobulins. Although not identical, the immunosignatures of the two species partially overlapped with our analyses. The immunoprofiling discrepancies between the two studies may be explained by the different doses and compositions of vaccines used. However, most of the proteins giving the highest reactivity in protein array in this study have been previously shown to be immunogenic in mice and humans. Previous reports of antigen-specific responses upon vaccination with different OMV-based vaccines, including *MeNZB* which corresponds to the OMV component of 4CMenB, showed that Omp85, PorA, PorB, RmpM, NspA (similar to results in this study) and FetA, FbpA, and LOS were immunogenic antigens upon vaccination^11–17^. By prioritizing OMV protein antigens, we did not explore the contribution of membrane-bound LOS into vaccine-induced protection, despite the fact that the percentage of residual LOS in OMV after detergent treatment represents roughly the 5–8% of the original one^53^. Antibodies reactive to RmpM have been identified in sera of different OMV-vaccinees^11,13,15^, but once purified they did not show any bactericidal activity^54^. Nevertheless, the recent study carried out with human recipients of the *MenPF1* vaccine pointed out that RmpM immunogenicity correlates with log(SBA) titers in five different meningococcal isolates^55^. Omp85, a highly conserved protein essential for the proper positioning and folding of OMPs in the bacterial membrane, has been hypothesized to be the reactive 80-kDa protein observed in immunoblots probed with antisera from individuals vaccinated with the Norwegian OMV^16,56^. Despite its immunogenicity, when Omp85 was overexpressed in meningococcal OMV it failed to induce higher bactericidal antibodies compared to wild-type vesicles in mice^57^. Thus, Omp85 appeared to be poorly bactericidal on its own. Nevertheless, an increase in functional activity has been described when Omp85 sera were combined with antisera against other OMPs, suggesting a putative synergy between antibodies, that can also occur following 4CMenB vaccination^56^. Unanticipated outcomes of the protein array analysis were MtrE and BamE, about which relatively little is known for meningococci. MtrE has been studied as a vaccine candidate in gonococci and it was recently shown that antibodies able to recognise gonococcal MtrE were induced by 4CMenB vaccination^58^. In our study, although identified as immunogenic in 4CMenB antisera, anti-Omp85, -RmpM, and -MtrE were not functional in the bactericidal assay. Importantly, the antibodies against PorA, PorB, OpcA, and NspA were able to kill the OMV-indicator strains in a strain-specific manner, suggesting their role in the immunogenicity of OMV. Furthermore, through the SBA analysis on OpcA deletion mutant strain and PorB-competition experiments we showed that antibodies targeting OpcA and PorB in mice and humans contribute to the bactericidal activity induced by the OMV vaccine component. In other studies, antibodies to OpcA have been shown to contribute to the bactericidal activity induced in humans from the Norwegian^16,17^ as well as other investigational OMV vaccines^55,59,60^. OpcA is well conserved, and its expression is phase-variable. In this study OpcA was identified as an additional OMV antigen responsible for the killing of 8 out of 12 OMV-specific isolates where *opcA* gene is largely phase ON and highly expressed. Similarly, the OMV of the Norwegian vaccine-elicited antibodies that were able to kill uniquely those strains expressing high levels of OpcA, while they failed against intermediate or low OpcA expressor isolates^17^. Therefore, considering that OpcA is conserved and its expression is phase-variable in all meningococcal strains^61^, anti-OpcA antibodies elicited following 4CMenB vaccination may contribute to killing of any meningococcal strain where OpcA is phase ON. The identification of PorB as an additional bactericidal antigen in 4CMenB was less expected, although old studies performed on sera of patients convalescent from meningococcal disease showed higher IgG levels against PorB than PorA, which once purified were bactericidal^62,63^. Moreover, while both native and recombinant PorB have been shown to elicit bactericidal antibodies in mice^64^, limited evidence has been reported of bactericidal PorB responses to OMV vaccines in humans, mainly using isogenic double mutant strains lacking PorA and OpcA^16,65,66^. Our studies, demonstrated that, while all OMV-indicator strains expressed comparable levels of the protein PorB3, only M07576 and M09929 were susceptible to PorB-dependent killing. Overall, the lack of bactericidal activity seems caused by a decreased surface accessibility of the respective epitopes, rather than sequence polymorphism. In line with these findings, Michaelsen and colleagues showed that the PorB3 porin is poorly accessible for the antibody binding on live meningococci, compared to the PorB2 counterpart. Moreover, the authors clarified that the reason behind this observation may lay in the short extracellular loops and long carbohydrate chains of LOS molecules expressed by these strains^67^. Therefore, we can speculate that the underlying reasons for the different SBA outcomes observed in our analysis may reside in an LOS shielding effect. Lastly, Neisserial surface protein A was identified as another protective antigen contained within OMV from mice immunogenicity studies, confirming previous findings about its ability to elicit bactericidal antibodies^68,69^ and to protect animals during in vivo meningococcal challenges^70,71^. In our study, only two strains, M07576 and M12898, were killed by NspA mouse antisera. The high level of identity of NspA primary sequence among isolates ruled out the involvement of protein variability in the different susceptibility to the killing observed, while flow cytometry analyses showed differences in NspA surface accessibility between the OMV-specific strains, as already observed for PorB. Several investigators have reported an inverse correlation between the amount of polysaccharide capsule and NspA surface accessibility, which might be related to the limited length of NspA surface-exposed loops and, therefore, a reduced bactericidal activity of anti-NspA antibodies in highly encapsulated isolates^68,72,73^. However, in our experiments with defined mutants lacking NspA we were not able to clearly ascribe the differential susceptibility to the killing with mouse or human sera to NspA expression or to its surface accessibility. Halperin et al. showed that recombinant NspA protein was immunogenic following a Phase I clinical trial, but it failed to provide protection against *N. meningitidis*. The authors hypothesized that the unfolded nature of the vaccine protein antigen, which may lack important conformational epitopes essential for the production of functional antibodies, might have contributed to such failure^74^. More recent data suggested that binding of human, but not mouse, complement factor H to NspA may impair elicitation of functional NspA antibodies in humans^75^. In our mouse immunization studies, the antigen was expressed in the outer membrane of the *E. coli* GMMA and successfully elicited bactericidal antibodies, probably because of its native folding.

Notably, among the whole repertoire of antigens displayed by the OMV, the proteins found immunogenic and protective in this study are all involved in the interaction with different host complement factors^76^, as also the case of the recombinant vaccine components fHbp and NHBA^77,78^. Therefore, the possibility to elicit antibodies against these virulence factors is pivotal for the complement-mediated killing of bacteria. The antibodies may also compete with the binding to complement inhibitors, thereby enhancing bacterial clearance in vivo, as previously suggested for fHbp^79^.

Interestingly, several real-world evidence has recently emerged that *MeNZB* and 4CMenB immunizations are associated with a moderate decrease in *Neisseria gonorrhoeae* infections in vaccinated subjects, indicating 30–40% vaccine effectiveness^80–82^. Considering that, at the genome level, *N. meningitidis* shares up to 80–90% homology with *N. gonorrhoeae*^83^ and that all the vaccine protein antigens, with the exception of NadA and PorA, are conserved in the gonococcus, it may be valuable to investigate the possible interspecies protection conferred by PorB, OpcA and NspA. Moreover, recent studies carried out in more than ten countries have shown that 4CMenB provides immunity against non-B meningococcal serogroups^83^. The protection has been mainly attributed to the four major vaccine antigens that, when expressed with sufficient similarity and density, can be targeted by vaccine-induced antibodies. Nevertheless, other, hitherto unidentified, OMV determinants may potentially contribute to the killing of non-B serogroups.

In conclusion, we have demonstrated that the inclusion of OMV antigens within 4CMenB is pivotal to enhance the breadth of coverage against a panel of non-vaccine-related strains, confirming the key role played by the vesicles within this multicomponent vaccine. Moreover, through the dissection of the OMV immunological repertoire, we were able to identify some non-PorA antigens involved in such broad protection and to demonstrate their protective role in the 4CMenB vaccine.

## Methods

### Study design

The purpose of this study was to elucidate the protective role of OMV antigens contained within the 4CMenB vaccine licensed for the active immunization against *N. meningitidis*. Human and animal sera were then used for this purpose.

The human infant serum samples used in this study were obtained via a Phase II clinical trial (V72P16, NCT00937521) conducted in multiple centers in the Czech Republic, Italy, Hungary, Chile and Argentina between July 2009 and November 2010 in accordance with Good Clinical Practices and according to the Declaration of Helsinki with approval of the protocol by ethics committees of participating centers. Parents or guardian of each participant have given their written informed consent to the study. The subjects, healthy infants aged approximately 2 months at the time of enrollment, received at 2, 3, 4, and 12 months of age four doses of different formulation: (i) rMenB + OMV (namely 4CMenB, 50 µg each recombinant protein + 25 µg OMV from NZ98/254 epidemic strains), (ii) rMenB + 1/4 OMV (50 µg each recombinant protein + 6.25 µg OMV), or (iii) rMenB alone (50 µg each recombinant protein). Sera samples used in this analysis are the anonymous pool of 25 subjects. Due to the limited amount available and the restrictions of informed consent forms the unique biological samples are not available.

All animal sera used in this study derived from mouse immunization experiments performed were performed at the GSK Animal Research Centre in Siena, Italy, in compliance with the ARRIVE guidelines, the current Italian legislation on the care and use of animals in experimentation (Italian Legislative Decree 116/92) and consecutive ministerial newsletter (Circolare Ministeriale 8/94), and with the GSK Animal Welfare Policy and Standards. The animal protocol was approved by the Animal Welfare Body of GSK Vaccines, Siena, Italy, and by the Italian Ministry of Health (Approval number AWB 2018_02, AWB2013_11 and AWB2017_04).

Four to six-weeks-old CD1 female mice (eight per group, Charles River) were immunized intraperitoneally on days 1, 22 and 36 with 8 µg of recombinant *E. coli* GMMA or OMV NZ98/254 and with 20 µg of recombinant proteins. Prior to immunization, samples were formulated with 3 mg/ml aluminum hydroxide as adjuvant in a volume of 200 µl each dose. Mouse sera were collected the day before the first immunization (day 0) and 2 weeks after the third immunization (day 50). To prepare 4CMenB and rMenB antisera CD1 female mice (8–10 per group), were immunized as above using 20 µg of each individual antigen (GNA2091-fHbp, NHBA-GNA1030, NadA) + 10 µg OMV or 20 µg of each individual antigens, respectively.

### Bacterial strains and culture conditions

*E. coli* and *N. meningitidis* strains used in this study are listed in Supplementary Table 2. *E. coli* strains were routinely grown in Luria-Bertani (LB) agar plates or medium at 37 °C. When required ampicillin, chloramphenicol, and kanamycin, were added to a final concentration of 100, 20 and 50 µg/ml, respectively. *N. meningitidis* strains were routinely cultured overnight on Gonococcus (GC) agar medium (Difco) with Kellogg’s supplement I or on chocolate agar plates (Biomérieux) at 37 °C in an atmosphere of 5% CO_2_. Liquid cultures were grown under the same conditions in Mueller Hinton (MH) broth plus 0.25% glucose. When required, erythromycin (5 μg/ml), kanamycin (150 μg/ml) or spectinomycin (50 μg/ml) were added to culture media.

### DNA manipulation and construction of plasmids

DNA manipulations were carried out using standard laboratory methods^84^ and all cloning steps were performed using the PIPE (Polymerase Incomplete Primer Extension) method^85^. For the expression of the meningococcal antigens considered in this study (Supplementary Table 3), the specific DNA fragments were amplified by PCR from MenB NZ98/254 genomic DNA using primers that amplified genes without their natural leader sequence (iPCR) (Supplementary Table 4). The presence of the leader peptide and location of its cleavage site were determined using Signal P 4.1^86^. Resulting iPCR were cloned into a pET21-LPOmpA expressing vector, a pET21 derivative plasmid carrying the sequence encoding the *E. coli* K-12 OmpA leader peptide (MKKTAIAIAVALAGFATVAQA), 6-histidine tag sequence (HHHHHH) and the FLAG tag (DYKDDDDK). The vPCR (vector-PCR) was amplified using different primers depending on the desired location of the Tag: HISFLAG Fw/OmpRev for C-terminal tags, while For-pet21/HisFlagRev-pet21 for N-terminal ones. Unpurified vPCR and iPCR DNA fragments were mixed in a 1:2 ratio and directly transformed into chemically competent Stellar cells (Takara). Cells were plated on LB containing 10 µg/ml of ampicillin and grown at 37 °C overnight (ON). Positive clones were selected by PCR analysis. Plasmid DNA was isolated (Supplementary Table 5), the correct nucleotide sequence of each plasmid was verified by sequence analysis and plasmids were used to transform BL21(DE3) Δ*tolR*^87^ or BL21(DE3) Δ*ompA*^88^ strains previously made chemically competent. Transformants were grown on LB plates containing 100 µg/ml of ampicillin. pET15 vector with OmpA leader sequence and without insert was instead used to transform BL21(DE3) Δ*tolR* and BL21(DE3) Δ*ompA* strains as a negative control.

### Purification of *E. coli* GMMA

The recombinant strains were pre-cultured over-day (6–7 h) in 5 ml of LB supplemented with ampicillin and growth at 37 °C, in a rotary shaker (180 rpm). Pre-cultures were then diluted 1:100 in 50 ml High Throughput Medium Complex (HTMC) (3% yeast extract, 1.5% glycerol, 40 mM KH_2_PO_4_, 90 mM K_2_HPO_4_, 2 mM MgSO_4_·7H_2_O, pH 7.4) + ampicillin and grown ON at 20 °C, 160 rpm. The cultures were then centrifuged 10 min at 1800×*g* at 20 °C, the supernatant was discarded to remove GMMA Empty and the pellet was resuspended in 60 ml of fresh HTMC supplied with the antibiotic. The expression of the recombinant proteins was induced with iso-propyl β-d-1-thiogalactopyranoside (IPTG) (Sigma-Aldrich) at a final concentration of 1 mM. Cultures were then incubated for 6 h at 37 °C, 180 rpm. Induced bacterial samples were collected to verify meningococcal protein expression in total as well as in soluble protein fractions. The cultures were clarified by centrifugation for 20 min at 3000 × *g* and the culture media were filtered through a 0.22-µm pore size filter (Millipore). The supernatants were subjected to high-speed centrifugation at 119,000 × *g* for 2 h at 4 °C (Beckman Coulter Optima Ultracentrifuge) and the pellets containing the GMMA were washed with phosphate buffer saline (PBS), ultracentrifuged again as above and finally resuspended in PBS after being filtered with 0.22-µm filter. GMMA total protein content was quantified through the Lowry assay (DC Protein Assay, Bio-Rad) following the manufacturer’s instructions.

### Purification of recombinant proteins

A single colony of recombinant *E. coli* BL21(DE3) Δ*tolR* or *E. coli* BL21(DE3) Δ*ompA* strains expressing the various meningococcal antigens were inoculated in 5 ml of LB added with ampicillin and grown for 7 h at 37 °C, 160 rpm. The cultures were then diluted 1:100 in 75 ml of HTMC medium + ampicillin, and they were grown ON at 20 °C, 160 rpm. The following day, protein expression was induced by the addition of 1 mM IPTG and cultures were grown at 20 °C, 160 rpm for 24 h.

For protein purification, bacterial pellets were resuspended in equilibration buffer (50 mM NaH_2_PO_4_ and 300 mM NaCl, pH 8.0) supplemented with a tablet of protease inhibitor cocktail (cOmplete, EDTA-free, Roche), each 50 ml of equilibration buffer, before mechanical lysis by sonication (amplitude 65%, 30 s ON/OFF, total 30 min). Alternatively, bacterial pellets were resolubilized in B-PER Bacterial Protein Extraction Reagent (B-PER, Thermo Fisher, 1 g/10 ml) or Cell-Lytic Express (Sigma-Aldrich) and incubated for 1 h at room temperature (RT) and periodically mixed. The unbroken cells and bacterial debris were removed by centrifugation and the recombinant His_6_-tagged meningococcal proteins were purified from the supernatant by IMAC (immobilized metal-ion-affinity chromatography) purification using cobalt-based chelating columns (Hitrap TALON Crude; Merck) according to the manufacturer’s instructions. Columns were washed with equilibration buffer, and proteins bound to the resin were then eluted with imidazole gradient (from 0 mM to 250 mM imidazole) in equilibration buffer. After this first purification step, sample purity was assessed on an analytical size-exclusion by Ultra Performance Liquid chromatography (SE-UPLC) using BEH200 4.6 × 300 mm columns (Waters) and using as running buffer 10 mM NaH_2_PO_4_, 400 mM (NH_4_)_2_SO_4_, pH 6.0. Samples whose purity was higher than 80% were dialyzed in PBS or loaded in a desalting column (GE Healthcare) and eluted in PBS. When sample purity was lower than 80%, samples were further purified by preparative size-exclusion chromatography (SEC) (Superdex 75/200) columns (HiLoad, GE Healthcare) in PBS. Endotoxin quantity was detected using LAL-test (Charles River) and when endotoxin levels were higher than >0.5U/µg, another purification step was performed with cHT (Bio-Rad) following the manufacturing protocols. Finally, proteins were analyzed by SDS‐PAGE followed by Coomassie blue staining (Giotto), and SE-UPLC to evaluate sample purity. Protein content was quantified by the BCA assay (Thermo Fisher).

The recombinant PorA and PorB proteins were instead purified from inclusion bodies. PorA bacterial pellets were dissolved in Guanidine 6 M, 50 mM Tris-HCl, pH 8.5, whereas PorB bacterial pellets were lysed in 8 M Urea, 50 mM Tris-HCl, pH 8,0. Undissolved particles were removed by centrifugation at 12,000 × *g* for 30 min at 4 °C. The solubilized inclusion bodies were concentrated in an Amicon concentrator, 10 kDa cutoff (Millipore). Then samples were diluted into a refolding buffer (200 mM CAPS, 400 mM NaCl, 50 mM Tris-HCl, 0.3% Lauryl-N,N-dimethylamine-N-Oxide [LDAO], pH 11). The mixtures were stirred ON at 4 °C and the pH was adjusted to 8.0 with 1 N HCl. The refolded porins were loaded onto an IMAC column (HisTrap HP, GE Healthcare), washed with PBS + 0,1% LDAO (PBSL) and eluted with PBSL containing 300 mM imidazole. The samples were concentrated with Amicon (10 kDa cutoff) and then loaded onto an S-300 Sephacryl column (GE Healthcare) and the protein was eluted in 0,1% PBSL. Sample purity was determined on an SE-HPLC (Superdex 200 5/150 GL) using as running buffer PBSL and the endotoxin content quantified as above stated. Finally, protein quantity was determined using Nanodrop (Thermo Fisher).

### Protein-microarray generation and immunoprofiling experiments

In total, the vesicles protein chip encompassed 26 recombinant *E. coli* GMMA, two GMMA empty, OMV from NZ98/254 (1 mg/ml or 0.5 mg/ml in 20% glycerol) and the 3 rMenB proteins (0.5 mg/ml in 40% glycerol), while the recombinant protein chip contains proteins spotted at 0.5 mg/ml in 40% glycerol. Controls consisted of 8 serial twofold dilutions of mouse and human IgG (from 0.5 mg/ml to 0.004 mg/ml in 40% glycerol), proteins expressed and purified from *E. coli* following the same expression and purification protocol, but originating from different pathogens other than MenB (0.5 mg/ml in 40% glycerol) and PBS + 40% glycerol spots. All samples were printed randomly in replicates onto ultra-thin nitrocellulose-coated glass slides (FAST slides; Maine Manufacturing). Printing was performed with the ink-jet spotter Marathon Argus (Arrayjet) (200 pl each spot) in a cabinet with controlled temperature and humidity (18 °C and 50–55%, respectively). To assure the efficiency and the reproducibility of protein immobilization some test slides were probed with: (1) anti-FLAG antibodies (Sigma-Aldrich, cat# F7425) 1:5000, (2) mouse anti-His_6_ tag polyclonal antibodies (Thermo Fisher, cat# 37-2900) 1:1000, (3) anti-GMMA Empty mouse sera (1:2000), and followed by detection with an AlexaFluor 647-conjugated anti-rabbit or anti-mouse IgG secondary antibody (Jackson Immunoresearch, cat# 111-605-046, cat# 115-605-174)—1:800. Preliminary experiments with animal and human sera showed that 1:1000 dilutions of sera corresponded to the best signal to noise ratio.

For sera profiling experiments slides were saturated 1 h with BlockIt (ArrayIt). Sera samples were then diluted 1:1000 in Block-It buffer and incubated for 1 h prior to undergo two washes with Tween 0.1% in PBS (TPBS). Slides were incubated with 1:800 diluted AlexaFluor 647-conjugated anti-mouse or anti-human IgG secondary antibody (Jackson Immunoresearch, cat# 115-605-174, cat# 309-605-008) another hour. After two final washes with TPBS and one in PBS, slides were rinsed in de-ionized water and dried. Fluorescence images were obtained using Power scanner (Tecan Trading) and the 16-bit images were generated with PowerScanner software v1.2 at 10 μm/pixel resolution. Spot fluorescence intensities were determined using ImaGene 9.0 software (Biodiscovery Inc.) and microarray data analysis was performed using *in-house* developed R scripts. For each protein the fluorescence intensity data of the replicates was subtracted by local background values surrounding each spot and averaged, hence resulting in a single data point per sample named Mean Fluorescence Intensity (MFI). Signals were considered positive when their MFI were greater than 5000, corresponding to the MFI of control protein spots after detection with fluorescent-labeled anti-mouse antibodies, plus ten times the standard deviation. All obtained MFI scores were then ranked in four categories: (1) high reactivity; MFI ≥ 30,000; (2) medium reactivity; 15,000 ≤ MFI > 30,000; (3) low reactivity; 5000 ≤ MFI > 15,000; (4) no reactivity; MFI < 5000.

### Serum bactericidal assay

For serum bactericidal activity against *N. meningitidis* strains bacteria were grown at 37 °C in MH broth plus 0.25% glucose until early log phase (OD of ∼0.25) and then diluted in Dulbecco’s saline phosphate buffer (Sigma) with 0.1% glucose and 1% BSA to a working dilution of 10^4^–10^5^ CFU/ml. The final assay mixture contained serial twofold dilutions of sera (previously heat inactivated for 30 min at 56 °C) and 25% complement represented by pooled baby rabbit serum (Cedarlane) or exogenous human complement deriving from donors with no detectable intrinsic bactericidal activity. Human complement source used in the study was obtained according to Good Clinical Practice and to the declaration of Helsinki. Patients have given their written consent (MENB REC 2ND GEN-074 (V72_92)). Serum bactericidal titers were defined as the serum dilution resulting in a 50% decrease in the number of viable colonies after 60 min of incubation relative to baseline colony counts at time zero^89^. Competitive SBAs were performed by adding recombinant protein to the serum samples to a final concentration of 1500 μg protein/ml serum, incubating overnight at 4 °C, and then diluting and testing the sample in SBA as above. Sera pre-treated with equal protein volume of PBS were used as controls for mimicking the “dilution-effect”.

### Construction of meningococcal deletion mutants

Plasmids for the allelic replacement of *opcA* and *nspA* coding sequences were obtained from^90^. PorA knockout was generated by replacing the promoter region and nucleotides 1–512 of *porA* coding sequence with a negative selection cassette (*tetR-sacB*) from pJJ260 vector^91^ and a spectinomycin resistance cassette through homologous recombination. About 500 basepair upstream and downstream to the target *locus* were PCR amplified using porAkoUPF/porAkoDOR from NZ98/254 genomic DNA and inserted into pET15 vector by PIPE cloning. The resulting plasmid was used as template for the vPCR using porAkoUPR and porAkoDOF. In a second step, the iPCR positive-negative selection cassette (*tetR-sacB-specR*) was inserted between the recombination fragments (vPCR). Finally, the obtained construct was PCR amplified using porAkoUPF and porAkoDOR, purified and used for the transformation. Strains, primers and plasmids used are listed in Supplementary Tables 2, 4, and 5, respectively. Linearized plasmids were used to transform *Neisseria meningitidis*. Four to seven colonies of a freshly grown ON plate were resuspended in 100 μl of PBS, mixed with 5 μg of DNA and spotted onto GC agar plates, allowed to dry, and incubated for 6 h at 37 °C, 5% CO_2_. Spots were then plated onto selective GC agar plates. Single colonies were further selected and amplified onto another plate with antibiotics. Correct clones were confirmed by PCR and western blotting analysis.

### Flow cytometry assay

The ability of mouse polyclonal sera to bind antigen present onto the surface of live meningococci grown in the same conditions as for the SBA assay was determined using flow cytometry analysis. Bacteria grown until early log phase were incubated for 1 h at RT with antibodies of interest (1:100). Primary antibody binding was detected using an Anti-mouse (whole-molecule) FITC-conjugated antibody (Sigma-Aldrich, cat# F9006) at a 1:100 dilution after 30 min of incubation. Bacteria were fixed with a 0.5% formaldehyde solution in PBS buffer for 1 h. Bacterial fluorescence was recorded with BD FACS CANTO II (BD Bioscience), acquiring 10,000 events, and data were analyzed using Flow-Jo v.10.8.1 (FlowJo, LLC). Bacteria incubated with secondary antibodies were used as negative controls. A figure exemplifying the gating strategy is provided in the Supplementary Information (Supplementary Fig. 10).

### SDS-PAGE and western blot analyses

*N. meningitidis* total crude lysates were prepared from strains grown in the same conditions as for the SBA assay to an OD of 0.25. Bacteria suspensions were then centrifuged 15 min at 1800 × *g* and the pellets resuspended in lysis buffer (5% SDS, 100 mM Tris-HCl, pH 8, 50 mM DTT, and protease inhibitor mixture (Roche)) to a final concentration of 0.007 OD/µl. In total, 10 µl of each meningococcal lysate (0.07OD/lane), recombinant meningococcal antigens (2 µg/lane or 1 µg/lane), *E. coli* GMMA (10 or 15 µg/lane, western blot or SDS-PAGE, respectively) and OMV NZ98/254 (10 µg/lane) were prepared in 4× NuPAGE LDS Sample Buffer (Thermo Fisher) and 10× NuPAGE Sample Reducing Agent (Thermo Fisher). *E. coli* cell lysates used to verify meningococcal protein expression were prepared from bacteria grown in LB. Five hundred microliters were removed from induced cultures and pelleted in a bench-top centrifuge 10 min at 9000 × *g*. Pellets were resuspended in 300 µl of B-PER (Thermo Fisher) and mix for 1 h at RT. The lysed solutions were added with 600 µl of binding buffer (20 mM NaPO_4_, 300 mM NaCl, 10 mM imidazol, pH = 8). Sixty microliters of the mixture, corresponding to the total fractions, were added with 20 µl of 4× NuPAGE LDS Sample Buffer + 10× Sample Reducing Agent. The remaining lysed solutions were centrifuged 20 min at 9000 × *g*. Sixty microliters of the supernatant, corresponding to the soluble fractions, were mixed with 20 µl of 4× NuPAGE LDS Sample Buffer + 10× Sample Reducing Agent and 12 µl of *E. coli* total and soluble fractions were loaded in each lane. All samples were boiled 10 min and separated by SDS-PAGE on NuPAGE Novex 4–12% Bis-Tris Protein Gels in MES 1× (Life Technologies). Gels were stained with ProBlue Safe Stain (Giotto Biotech). For western blot, gels were transferred to nitrocellulose membranes using iBlot Dry Blotting System (Invitrogen). After 1 h saturation with 5% powdered milk (Sigma-Aldrich) in TPBS, the filters were incubated with specific primary antibodies (specific mouse antisera 1:2000; mAb L3,7,9 from NIBSC cat# 01/412 1:2000, anti-FLAG from Sigma-Aldrich, cat# F7425, 1:5000) in 3% powdered milk in TPBS and incubated for 1 h. A horseradish peroxidase-conjugated anti-mouse or anti-rabbit IgG antibody (Thermo Fisher, cat# 62-6520, cat# 65-6120, diluted 1:2000) and the Pierce ECL Western Blotting Substrate (Thermo Fisher) were used according to the manufacturer’s instructions. ChemiDoc Touch (Bio-Rad) was used for data acquisition.

For quantification of heterologous protein expression, different amounts of GMMA were loaded onto 4–12% SDS-polyacrylamide gels along with increasing concentrations of the corresponding purified recombinant protein used as standard. The images were acquired using ChemiDoc (Bio-Rad) and analyzed with ImageLab v6 (Bio-Rad). The amount of recombinant antigens expressed in the GMMA was estimated by densitometry analysis of each lane using the formula: (µg of antigen expressed in GMMA/µg of total protein loaded) × 100. Novex Sharp Pre-stained Protein Standard (Thermo Fisher) was used as a protein ladder in all gels and western blots.

All blots and gels derived from the same experiment, and they were processed in parallel.

### Whole-genome sequencing and bioinformatic analysis

*N. meningitidis* genomic DNA from the 12 strains reported in Table 1 was extracted from cell suspensions of ON culture plates using the GenElute Bacterial Genomic DNA Kit (Sigma). Meningococci were sequenced using the Illumina HiSeq platform and read sequences were assembled using Spades, version 3.13, with default parameters. The resultant assemblies were uploaded to the internal *Neisseria* PubMLST database that was used for the typing of our loci of interest. Nucleotide sequences of the 30 OMV-loci were extracted from the genomes with genome comparator functionality, translated though EMBOSS transeq tool. Amino acid sequences were aligned, and identity percentage has been calculated using the NEEDLE tool. The percentages of amino acid identity (range and median) with respect of NZ98/254 were calculated through an R script developed internally.

### Enzyme-linked immunosorbent assay

Serum IgG titers directed to OMV-specific antigens were evaluated by enzyme-linked immunosorbent assay (ELISA). Ninety-six-well Maxisorp plates (Nunc, Thermo Fisher Scientific) were coated with 1 µg/ml of purified recombinant antigens in a buffer of 0.115% Na_2_HPO_4_, 0.02% KCl, 0.02% KH_2_PO_4_, 0.8% NaCl, pH 7.4 and incubated ON at 4 °C. Plates were then washed three times with 0.05% Tween-20 in PBS 0.074 M and blocked with 250 µl/well of 2.7% polyvinylpyrrolidone (Sigma-Aldrich) for 2 h at 37 °C. Each incubation step was followed by triple washes in 0.05% Tween-20 in PBS 0,074 M. Serum samples were diluted in 1% BSA (Bovine Serum Albumin) + 0.05% Tween-20 in PBS 0.074 M buffer, transferred to coated-blocked plates and twofold serially diluted, then incubated 2 h at 37 °C. One hundred microliters of 1:2000 alkaline phosphatase-conjugated goat anti-mouse IgG (Sigma-Aldrich, cat#A3562) diluted in 1% BSA in 0.05% Tween-20 in PBS 0.074 M were added to each well and left for 90 min at 37 °C. Bound alkaline phosphatase was visualized by the addition of 100 µl/well of p-nitrophenyl phosphate (Sigma-Aldrich) and incubation for 30 min at room temperature (RT). Then 100 µl of 4 N NaOH were added to each well and plates were analyzed at a dual wavelength of 405/620–650 nm in a microplate spectrophotometer (SLT Spectra, Tecan) in order to subtract the background. Antibody titers were quantified as the dilution of serum that gives an absorbance of 0.4 OD. This value represents the average of the preimmune sera (negative control) multiplied four times, and values major than 0.4 OD are considered positive.

Results were analyzed with GraphPad Prism Software 9 using *t* Student test to calculate statistical significance (*P ≤ 0.05; **P ≤ 0.01; ***P ≤ 0.001, ****P ≤ 0.0001).

### Trademarks

*Bexsero* is a trademark owned by or licensed to the GSK group of companies. *Trumenba* is a trademark owned by Pfizer. *VA-MENGOC-BC* is a trademark of the Finlay Institute, Cuba. *MenBvac* is a trademark of the Norwegian Institute of Public Health. *MeNZB* is a trademark of Novartis.

### Reporting summary

Further information on research design is available in the Nature Research Reporting Summary linked to this article.

## Supplementary information

REPORTING SUMMARY

Supplementary Material

## Data Availability

Data supporting the findings of this study are available on request from the corresponding author. The data discussed in this publication have been deposited in NCBI’s Gene Expression Omnibus and are accessible through GEO Series accession number GSE224348. The sequence reads of the *N. meningitidis* genome DNA are publicly available at National Center for Biotechnology Information, NCBI: accession number PRJNA930150.

## Code availability

Custom scripts developed in R are available on the GitHub repository: https://github.com/mbodini/CodeFor4CMenBvaccine_crosscoverage_paper.
