# Supplementary material for: OpcA and PorB are novel bactericidal antigens of the 4CMenB vaccine in mice and humans
Source: NPJ Vaccines. 2023 Apr 12;8:54. doi: 10.1038/s41541-023-00651-9 (PMC10097807; doi:10.1038/s41541-023-00651-9)
Supplement: Supplementary file 1 — REPORTING SUMMARY [file 41541_2023_651_MOESM1_ESM.pdf]

nature portfolio

Corresponding author(s): Erika Bartolini
Last updated by author(s): 31/01/2023

Reporting Summary

Nature Portfolio wishes to improve the reproducibility of the work that we publish. This form provides structure for consistency and transparency in reporting. For further information on Nature Portfolio policies, see our Editorial Policies and the Editorial Policy Checklist.

Statistics

For all statistical analyses, confirm that the following items are present in the figure legend, table legend, main text, or Methods section.

n/a	Confirmed	
☐	☒	The exact sample size (*n*) for each experimental group/condition, given as a discrete number and unit of measurement
☐	☒	A statement on whether measurements were taken from distinct samples or whether the same sample was measured repeatedly
☐	☒	The statistical test(s) used AND whether they are one- or two-sided<br>*Only common tests should be described solely by name; describe more complex techniques in the Methods section.*
☒	☐	A description of all covariates tested
☒	☐	A description of any assumptions or corrections, such as tests of normality and adjustment for multiple comparisons
☐	☒	A full description of the statistical parameters including central tendency (e.g. means) or other basic estimates (e.g. regression coefficient) AND variation (e.g. standard deviation) or associated estimates of uncertainty (e.g. confidence intervals)
☐	☒	For null hypothesis testing, the test statistic (e.g. *F*, *t*, *r*) with confidence intervals, effect sizes, degrees of freedom and *P* value noted<br>*Give P values as exact values whenever suitable.*
☒	☐	For Bayesian analysis, information on the choice of priors and Markov chain Monte Carlo settings
☒	☐	For hierarchical and complex designs, identification of the appropriate level for tests and full reporting of outcomes
☒	☐	Estimates of effect sizes (e.g. Cohen's *d*, Pearson's *r*), indicating how they were calculated

Our web collection on statistics for biologists contains articles on many of the points above.

Software and code

Policy information about availability of computer code

Data collection	A description and the version of all commercial instrument and softwares used for data acquisition are detailed in the Methods section of the article.
Data analysis	A description and the version of all commercial and open source softwares and code libraries used for the data analysis are detailed in the Methods section of the article.<br>Custom scripts developed in R are available on the GitHub repository: https://github.com/mbodini/ CodeFor4CMenBvaccine_crosscoverage_paper

For manuscripts utilizing custom algorithms or software that are central to the research but not yet described in published literature, software must be made available to editors and reviewers. We strongly encourage code deposition in a community repository (e.g. GitHub). See the Nature Portfolio guidelines for submitting code & software for further information.

Data

Policy information about availability of data

All manuscripts must include a data availability statement. This statement should provide the following information, where applicable:

- Accession codes, unique identifiers, or web links for publicly available datasets
- A description of any restrictions on data availability
- For clinical datasets or third party data, please ensure that the statement adheres to our policy

Data supporting the findings of this study are available on request from the corresponding author.
The data discussed in this publication have been deposited in NCBI's Gene Expression Omnibus and are accessible through GEO Series accession number GSE224348.
The sequence reads of the N. meningitidis genome DNA are publicly available at National Center for Biotechnology Information, NCBI: accession number PRJNA930150.

Human research participants

Policy information about studies involving human research participants and Sex and Gender in Research.

Reporting on sex and gender	The human serum used is an anonymous pool, no information about sex and gender
Population characteristics	The human sera used is an anonymous pool, no information about population characteristics of each participant
Recruitment	The human sera used is an anonymous pool
Ethics oversight	Human sera is an anonymous pool derived from V72P16, NCT00937521. No further approval required in addition to what was originally described in the following studies: A phase 2 randomized controlled trial of a multicomponent meningococcal serogroup B vaccine (I) and A phase 2 randomized controlled trial of a multicomponent meningococcal serogroup B vaccine, 4CMenB, in infants (II)

Note that full information on the approval of the study protocol must also be provided in the manuscript.

Field-specific reporting

Please select the one below that is the best fit for your research. If you are not sure, read the appropriate sections before making your selection.

☒ Life sciences ☐ Behavioural & social sciences ☐ Ecological, evolutionary & environmental sciences

For a reference copy of the document with all sections, see nature.com/documents/nr-reporting-summary-flat.pdf

Life sciences study design

All studies must disclose on these points even when the disclosure is negative.

Sample size	For animal studies we planned to use the minimum number of animals per experimental group to have a robust statistical significance of the data. Based on results previously obtained and taken into consideration the variability within the immunization studies, the minimum number of 8 animals per experimental group was chosen.<br><br>The human sera pool used in the study was empirically performed from 25 subjects.
Data exclusions	No data were excluded.
Replication	The immunization studies contain 8 animals per group and all the experiments performed with animal sera derived from pool, as stated in the manuscript.<br><br>All the experiments with animal/human sera were performed at least twice.
Randomization	Allocation of mice to the experimental groups was done on the basis of results previously obtained and taken into consideration the variability within the immunization studies.
Blinding	Investigators were not blinded to animal groups due to the nature of vaccine delivery.<br>Investigators were instead blinded on the 25 subjects used to perform the human sera pool used in the study.

Reporting for specific materials, systems and methods

We require information from authors about some types of materials, experimental systems and methods used in many studies. Here, indicate whether each material, system or method listed is relevant to your study. If you are not sure if a list item applies to your research, read the appropriate section before selecting a response.

Materials & experimental systems

n/a	Involved in the study
☐	☒ Antibodies
☒	☐ Eukaryotic cell lines
☒	☐ Palaeontology and archaeology
☐	☒ Animals and other organisms
☒	☐ Clinical data
☒	☐ Dual use research of concern

Methods

n/a	Involved in the study
☒	☐ ChIP-seq
☐	☒ Flow cytometry
☒	☐ MRI-based neuroimaging

Antibodies

Antibodies used	Anti-Meningococcal Immunotype L3,7,9 Monoclonal Antibody (NIBSC, 01/412)<br>ANTI-FLAG® antibody produced in rabbit (Sigma Aldrich, F7425)<br>Goat anti-Rabbit IgG (H+L) Secondary Antibody, HRP (Thermo Fisher, 65-6120)<br>6x-His Tag Monoclonal Antibody (4A12E4) (Thermo Fisher, 37-2900)<br>Goat anti-Mouse IgG (H+L) Secondary Antibody, HRP (Invitrogen, 62-6520)<br>Alexa Fluor 647 AffiniPure Goat Anti-Mouse IgG, Light Chain specific, (Jackson Immunoresearch, 115-605-174)<br>Alexa Fluor 647 AffiniPure Rabbit Anti-Human IgG, Fcγ fragment specific, (Jackson Immunoresearch, 309-605-008)<br>Alexa Fluor 647 AffiniPure Goat Anti-Rabbit IgG, Fc fragment specific, (Jackson Immunoresearch, 111-605-046)<br>Anti-Mouse IgG (whole molecule)–FITC antibody produced in goat (Sigma Aldrich, F9006)<br>Anti-Mouse IgG (whole molecule)–Alkaline Phosphatase antibody produced in goat (Sigma Aldrich, A3562)
Validation	N/A

Animals and other research organisms

Policy information about studies involving animals; ARRIVE guidelines recommended for reporting animal research, and Sex and Gender in Research

Laboratory animals	Four-six weeks-old CD1 female mice were used in the study
Wild animals	The study did not involve wild animals
Reporting on sex	All immunization studies were performed in female mice
Field-collected samples	The study did not involve samples collected from field
Ethics oversight	All animal sera used in this study derived from mouse immunization experiments performed were performed at the GSK Animal Research Centre in Siena, Italy, in compliance with the ARRIVE guidelines, the current Italian legislation on the care and use of animals in experimentation (Italian Legislative Decree 116/92) and consecutive ministerial newsletter (Circolare Ministeriale 8/94), and with the GSK Animal Welfare Policy and Standards. The animal protocol was approved by the Animal Welfare Body of GSK Vaccines, Siena, Italy, and by the Italian Ministry of Health (Approval number AWB2018_02, AWB2013_11 and AWB2017_04).

Note that full information on the approval of the study protocol must also be provided in the manuscript.

Flow Cytometry

Plots

Confirm that:

☒ The axis labels state the marker and fluorochrome used (e.g. CD4-FITC).

☒ The axis scales are clearly visible. Include numbers along axes only for bottom left plot of group (a 'group' is an analysis of identical markers).

☐ All plots are contour plots with outliers or pseudocolor plots.

☐ A numerical value for number of cells or percentage (with statistics) is provided.

Methodology

Sample preparation	Bacteria grown until early log phase were incubated for 1 h at RT (room temperature) with antibodies of interest (1:100). Primary antibody binding was detected using an Anti-mouse (whole-molecule) FITC-conjugated antibody (Sigma Aldrich) at a 1:100 dilution after 30 min of incubation. Bacteria were fixed with a 0.5% formaldehyde solution in PBS buffer for 1 h.
Instrument	Bacterial fluorescence was recorded with BD FACS CANTO II (BD Bioscience) acquiring 10,000 events.
Software	Data were analysed using Flow-Jo v.10.8.1 (FlowJo, LLC)
Cell population abundance	Not applicable for the analysis performed in the manuscipt
Gating strategy	Morphology (FSC-A vs SSC-A), Singles (SSC-W vs SSC-A) --> population of interest (FITC positive, SSC-A - Histogram overlay negative control vs tested sample).<br>Gating strategy available in the Supplementary Material

☒ Tick this box to confirm that a figure exemplifying the gating strategy is provided in the Supplementary Information.
