# Supplementary material for: OpcA and PorB are novel bactericidal antigens of the 4CMenB vaccine in mice and humans
Source: NPJ Vaccines. 2023 Apr 12;8:54. doi: 10.1038/s41541-023-00651-9 (PMC10097807; doi:10.1038/s41541-023-00651-9)
Supplement: Supplementary file 2 — Supplementary Material [file 41541_2023_651_MOESM2_ESM.pdf]

Supplementary Information

The PDF file includes:

Supplementary Figure 1. The vaccine immunotype L3,7,9 is expressed by most, but not all OMV-specific MenB strains.

Supplementary Figure 2. OMV antigens were expressed in GMMA obtained from different *E. coli* mutants engineered to hyperbleb.

Supplementary Figure 3. Four MenB antigens were expressed in *E. coli* cell lysates but they were not taken up by the corresponding GMMA.

Supplementary Figure 4. Thirteen different meningococcal proteins were obtained as recombinant antigens.

Supplementary Figure 5. Western blot analyses revealed that sera raised by mouse immunizations recognized the specific antigen expressed by different meningococci.

Supplementary Figure 6. ELISA measured the specific IgG titres in mice immunized with different *E. coli* GMMA and recombinant proteins.

Supplementary Figure 7. Estimation of the amount of the heterologous meningococcal antigen present in *E. coli* GMMA by comparative Western blots.

Supplementary Figure 8. FACS analysis of live *N. meningitidis* B strains using sera prepared against PorA, OpcA, PorB and NspA antigens.

Supplementary Figure 9. Uncropped and unprocessed Western blots used for Fig.4.

Supplementary Figure 10. Exemplification of the gating strategy used for FACS analyses of Fig. 4 and Supplementary Figure 8.

Supplementary Table 1. Rabbit SBA screening of polyclonal sera not reactive to OMV according to protein array analysis.

Supplementary Table 2. List of strains used in this study.

Supplementary Table 3. Meningococcal antigens cloned in this study.

Supplementary Table 4. List of primers used in this study.

Supplementary Table 5. List of plasmids used in this study.

References [1-4]

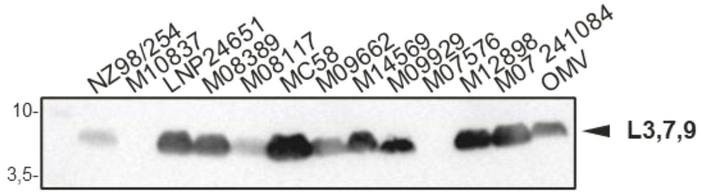

Supplementary Figure 1. The vaccine immunotype L3,7,9 was expressed by most, but not all, OMV-specific MenB strains. Whole cell lysates, prepared from bacteria grown in SBA-like conditions and OMV were resolved in SDS-PAGE prior to Western blotting. The membrane was probed with the immunotype specific monoclonal antibody L3,7,9. LOS bands are indicated by the arrow.

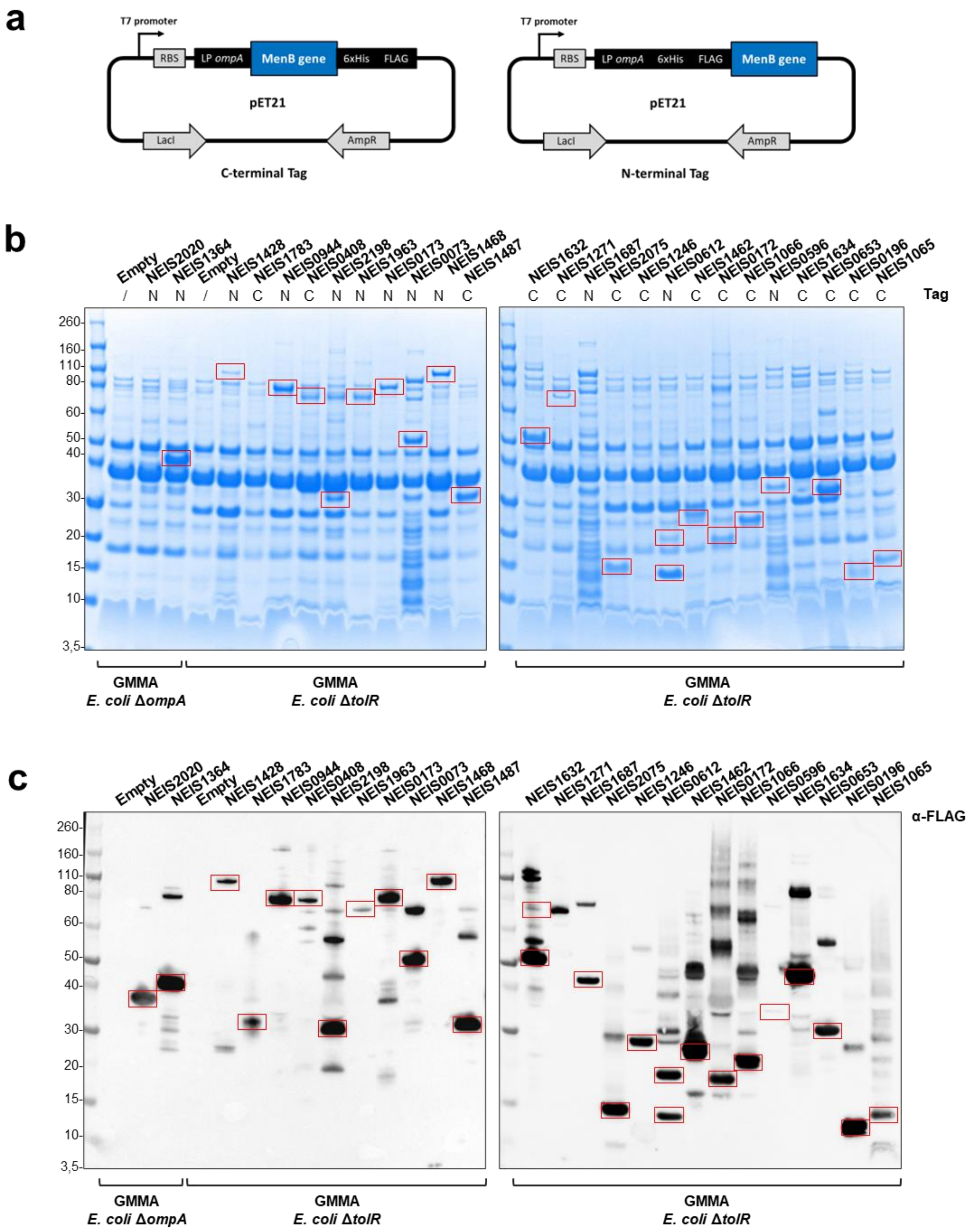

a
T7 promoter
RBS
LP ompA
MenB gene
6xHis
FLAG
pETG21
LacI
AmpR
C-terminal Tag
N-terminal Tag
b
Empty NEIS2020 NEIS1364 Empty NEIS1428 NEIS1783 NEIS0944 NEIS0408 NEIS2198 NEIS1963 NEIS0173 NEIS0073 NEIS1468 NEIS1487
/ N N / N C N C N N N N N C
NEIS1632 NEIS1271 NEIS1687 NEIS2075 NEIS1246 NEIS0612 NEIS1462 NEIS0172 NEIS1066 NEIS0596 NEIS1634 NEIS0653 NEIS0196 NEIS1065
C C N C C N C C C N C C C C
Tag
260 160 110 80 60 50 40 30 20 15 10 3,5
GMMA E. coli ΔompA
GMMA E. coli ΔtolR
GMMA E. coli ΔtolR
c
α-FLAG

Supplementary Figure 2. OMV antigens were expressed in GMMA obtained from different *E. coli* mutants engineered to hyperbleb. (**a**) Two different strategies were adopted for the cloning of meningococcal proteins into the expression vectors. The two vector maps differ only in the location of the tags, either carboxy- or amino-terminal (left and right maps, respectively). The vectors carry the IPTG-inducible T7 promoter, the leader peptide (LP) of the *E. coli ompA* and tag sequences composed of six-histidine (6xHis) and the FLAG. The cloning site of heterologous antigens is represented by the blue box labelled "MenB gene". RBS, ribosome binding site; LacI, *lac* repressor; AmpR, ampicillin resistance. Recombinant *E. coli* GMMA decorated with meningococcal antigens were analysed by (**b**) Coomassie blue stained SDS-PAGE and (**c**) Western blot probed with α-FLAG antibodies. Bands corresponding to recombinant antigens are boxed in red. Location of the tag sequence (N, N-terminal and C, C-terminal) as well as *E. coli* strains used for GMMA production are indicated.

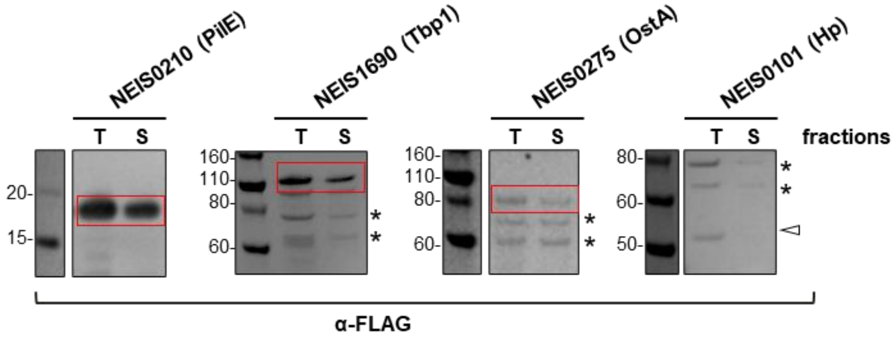

Supplementary Figure 3. Four MenB antigens were expressed in *E. coli* cell lysates but they were not taken up by the corresponding GMMA. Western blots probed with anti-FLAG antibodies were carried out on *E. coli* BL21 Δ*tolR* total (T) and soluble (S) cell-extract fractions expressing the specific MenB antigens with C-terminal tags. Bands corresponding to recombinant antigens are boxed. The open arrow indicates the expression of NEIS0101 exclusively in the total cell extract fraction. * indicates not specific band.

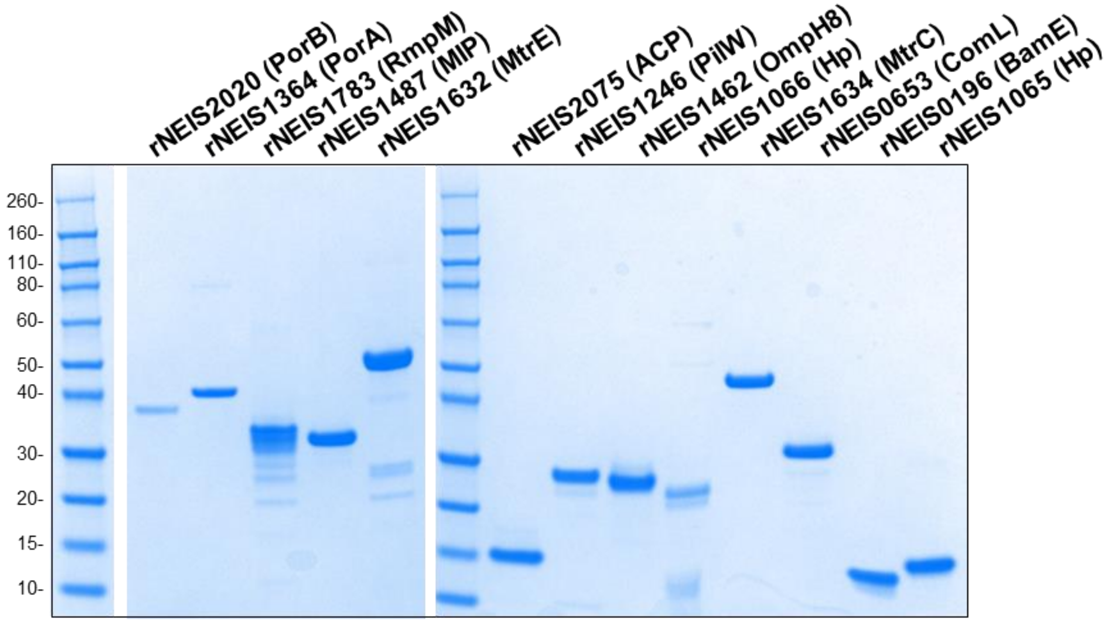

Supplementary Figure 4. Thirteen different meningococcal proteins were obtained as recombinant antigens. Equal amounts of meningococcal antigens (2 µg/lane, except for NEIS2020 (PorB) and NEIS1066 (Hp) 1 µg/lane) were resolved in a Sodium Dodecyl Sulphate - PolyAcrylamide Gel Electrophoresis and were stained with Coomassie blue.

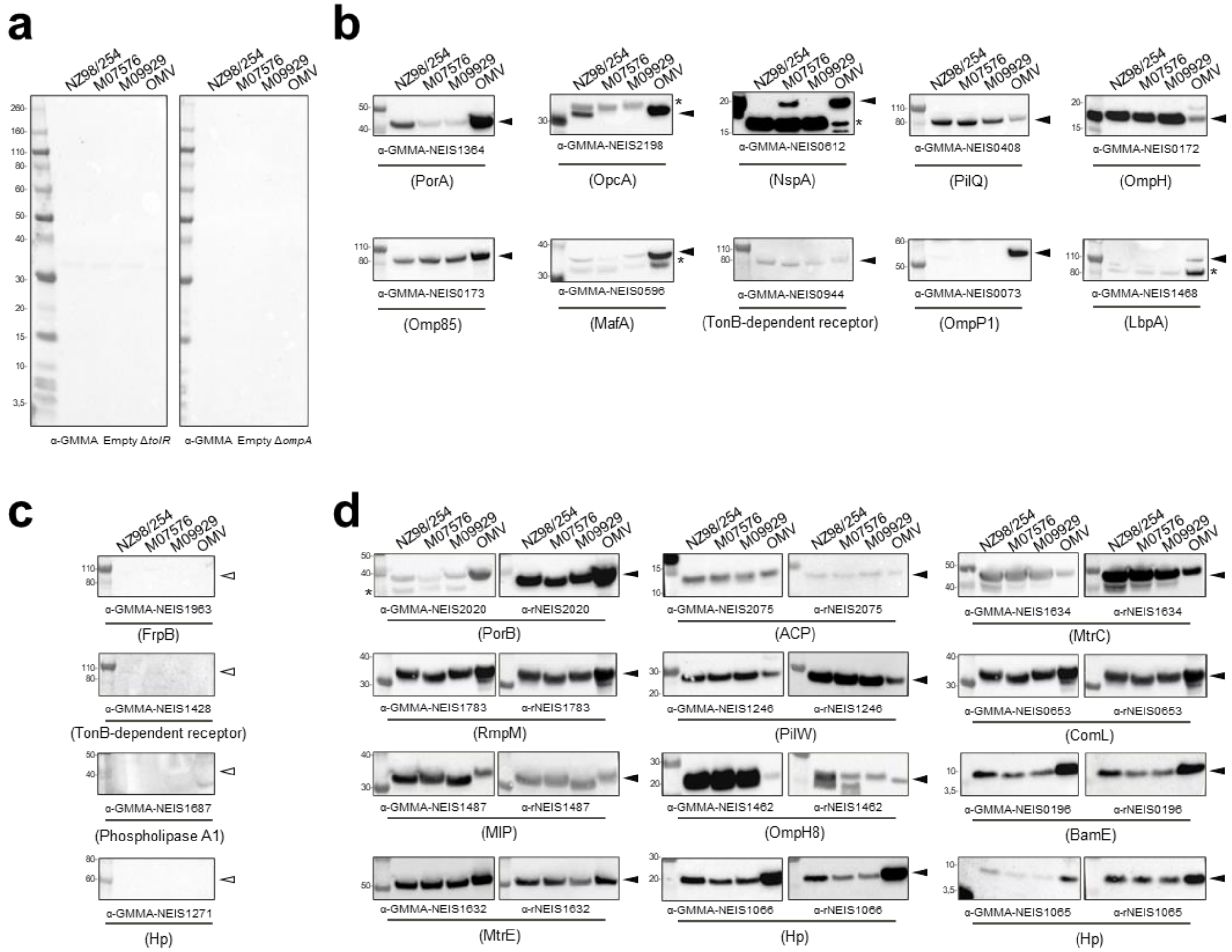

Supplementary Figure 5. Western blot analyses revealed that sera raised by mouse immunizations recognized the specific antigen expressed by different meningococci. Equal amounts of whole cell lysates as well as OMV were separated by SDS-PAGE prior to Western blotting. The specific antisera raised against: (**a**) GMMA Empty (negative control); (**b-c**) engineered GMMA and (**d**) coupled GMMA-recombinant used to probe each membrane are listed beneath each blot. All specific sera used for the analysis are pooled ($n = 8$). Protein bands corresponding to the expected molecular size of the protein are indicated by closed arrows. Open arrows indicate the molecular weight at which the specific-reactive band was expected. The asterisk pinpoints non-specific bands. Hp = hypothetical protein.

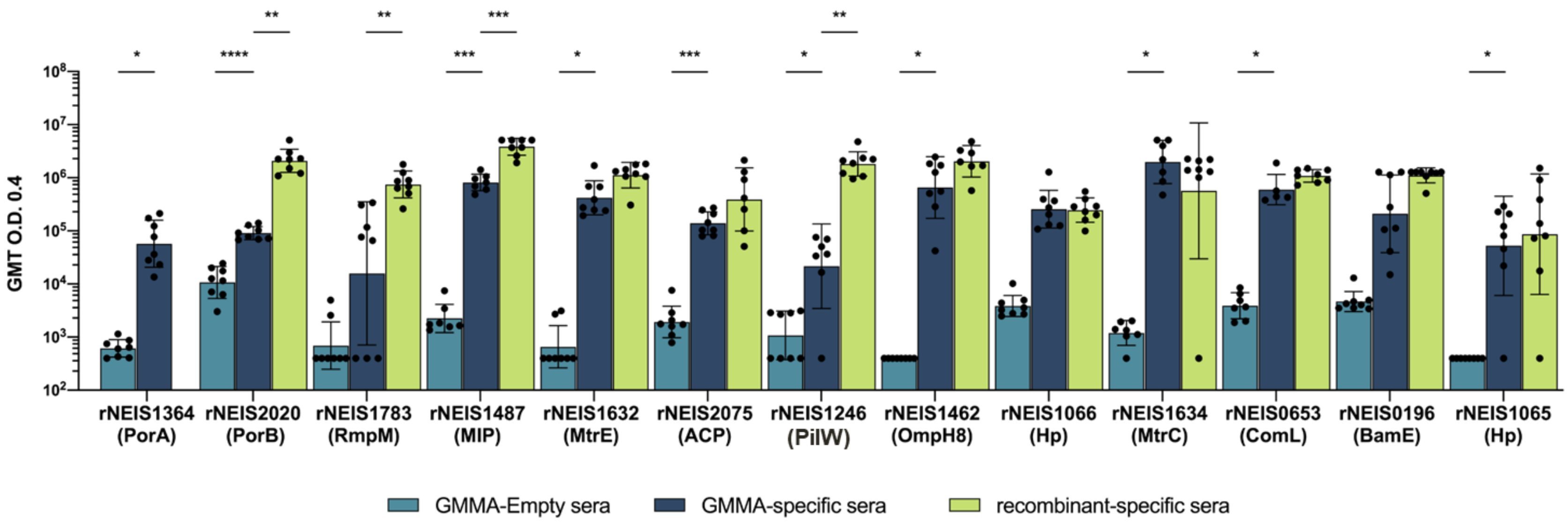

Supplementary Figure 6. ELISA measured the specific IgG titres in mice immunized with different *E. coli* GMMA and recombinant proteins. IgG titres were determined by ELISA using plates coated with the 13 recombinant antigens, listed on the X axis. Light blue bars indicate GMMA Empty antisera used as negative control, in particular *E. coli* GMMA Empty Δ*ompA* antisera was used for the plates coated with PorA and PorB antigens, whereas all the other coatings were probed with *E. coli* GMMA Empty Δ*tolR* antisera. Dark blue and green bars indicate the GMMA-specific and recombinant antigen-specific antisera which correspond to antigen used to coat the plates, respectively. Each dot represents the titre obtained from an individual mouse serum, while histograms indicate the geometric mean (GMT) value within each immunization, expressed as the reciprocal of the serum dilution yielding an OD value of 0.4. Error bars of each mouse group describe the geometric standard deviation. Statistical analysis was performed applying the t-student test and *p*-values ≤ 0.05 were considered statistically significant (* $p \leq 0.05$; ** $p \leq 0.01$; ***$p \leq 0.001$, **** $p \leq 0.0001$). Hp = hypothetical protein.

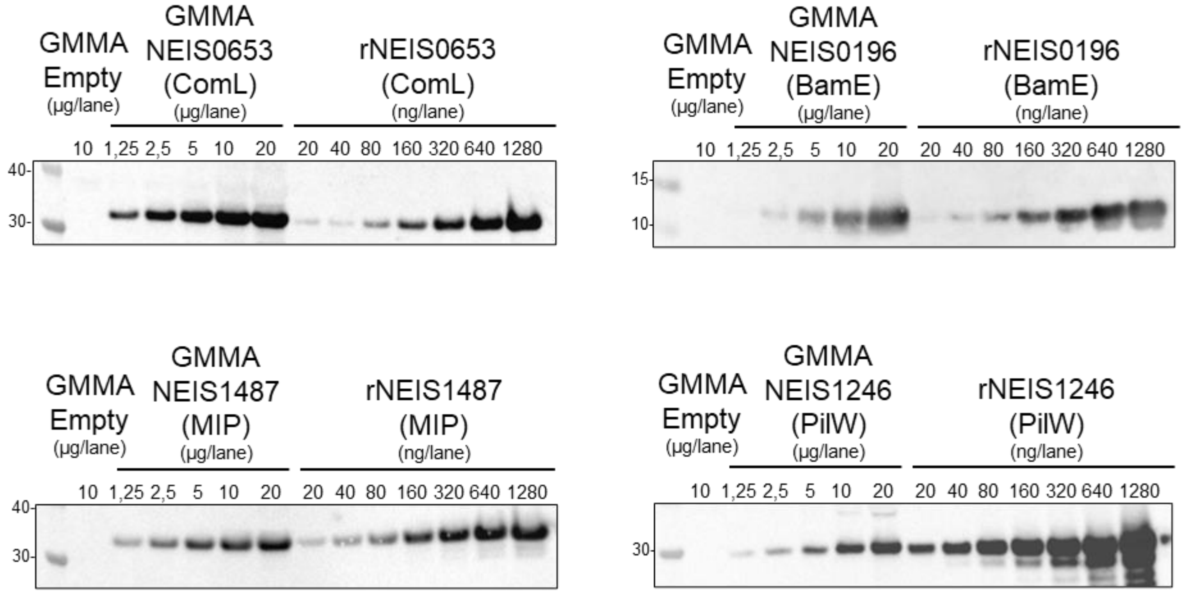

Supplementary Figure 7. Estimation of the amount of the heterologous meningococcal antigen present in *E. coli* GMMA by comparative Western blots. Different amount of GMMA were loaded onto 4-12% SDS-polyacrylamide gels together with increasing concentrations of the corresponding purified recombinant protein used as standard. Western blots were then probed with anti-FLAG antibodies. From the comparison of band intensities, the following percentages were determined: NEIS0653: 18%; NEIS0196: 3.2%; NEIS1487: 2.1%; NEIS1246: 0.27%.

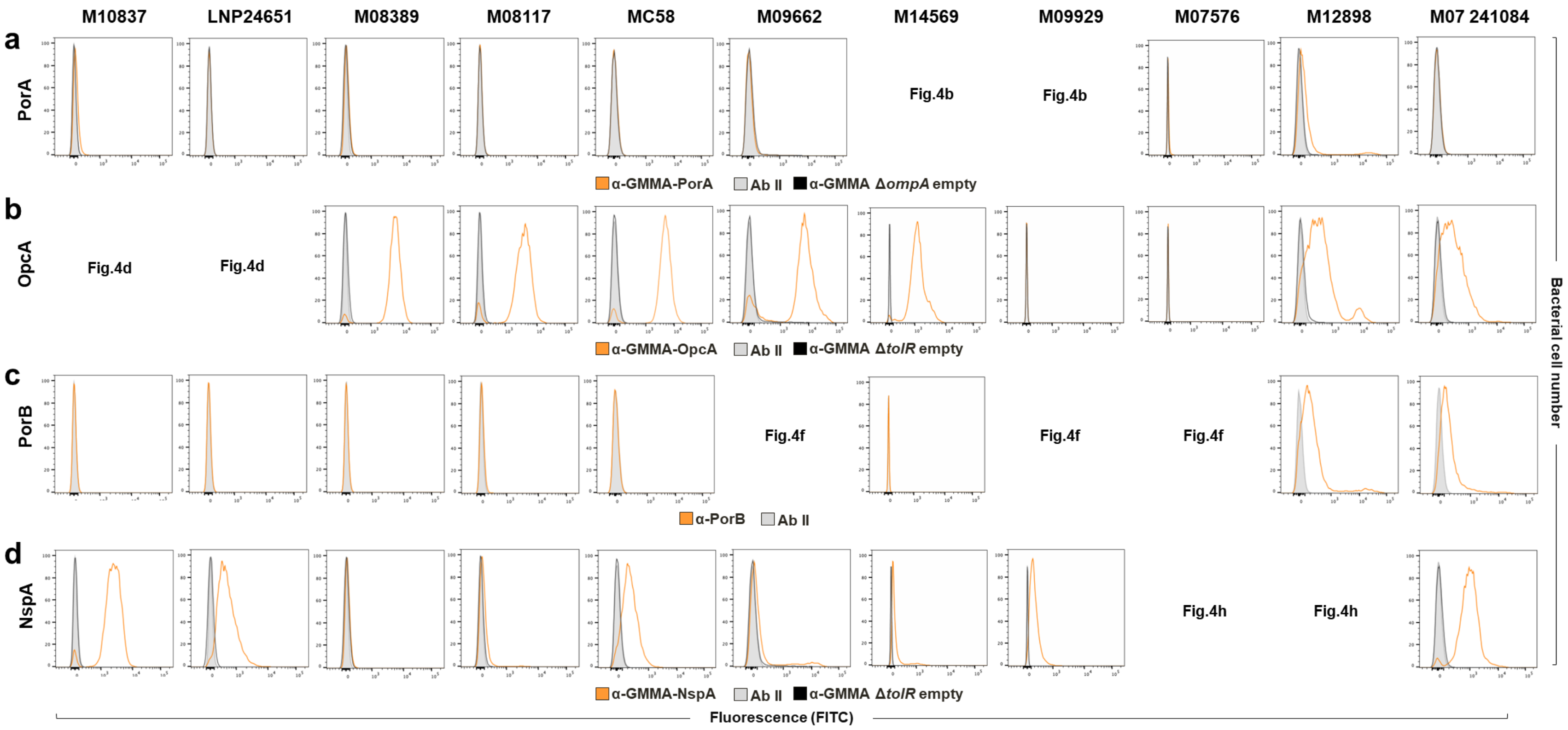

Supplementary Figure 8. FACS analysis of live *N. meningitidis* B strains using sera prepared against PorA, OpcA, PorB and NspA antigens. The binding of antisera raised by PorA (**a**), OpcA (**b**), PorB (**c**) and NspA (**d**) antigens was assessed by flow cytometry. Shaded grey profiles represent bacterial cells stained with secondary antibodies alone; non-shaded dark grey profiles indicate sera raised against GMMA empty Δ*tolR* and Δ*ompA* (negative controls); non-shaded orange profiles show the reaction with specific immune sera. Ab II: secondary antibody.

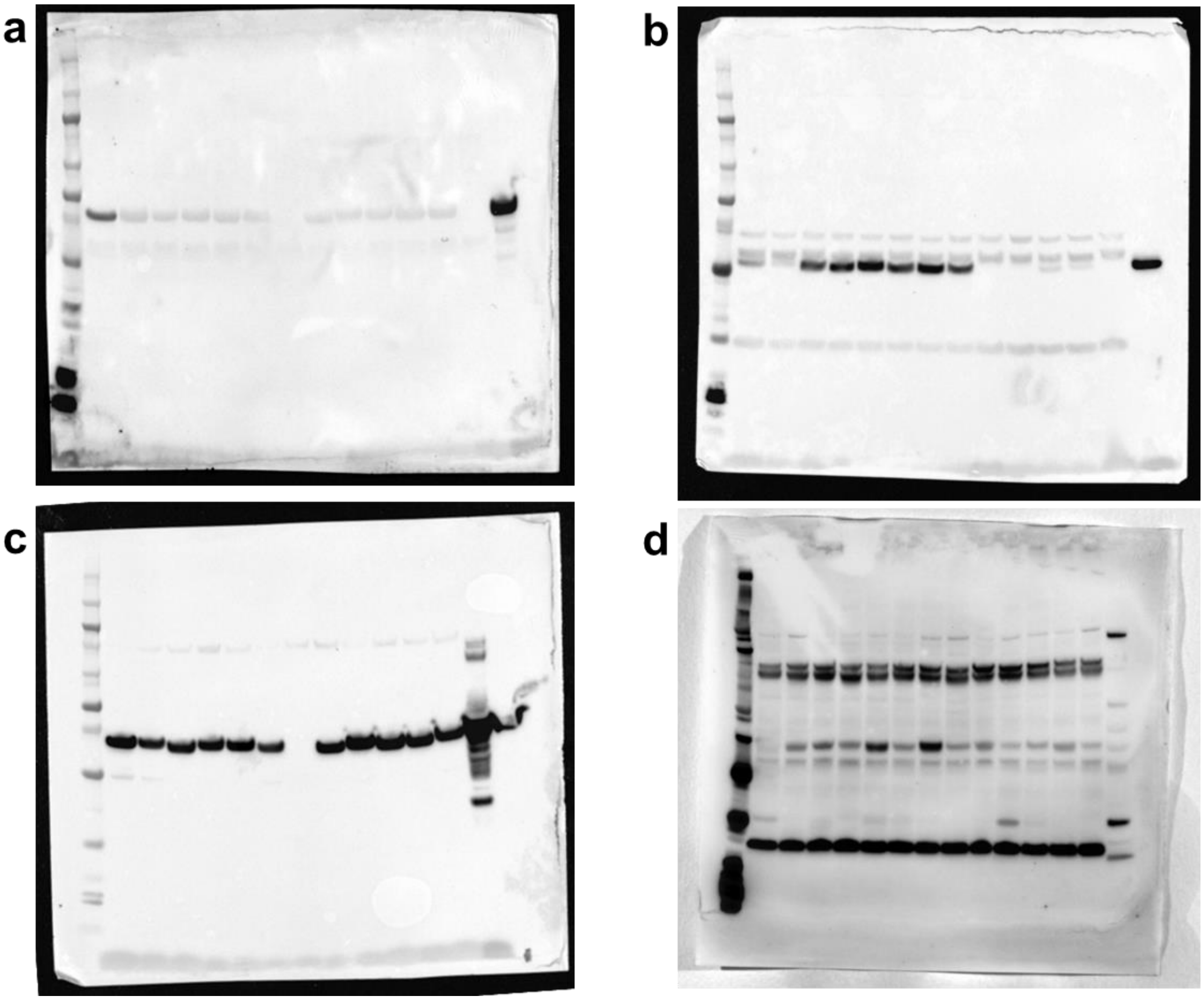

Supplementary Figure 9. Uncropped and unprocessed Western blots used for Fig.4. The uncropped and unprocessed scans probed with GMMA-PorA (**a**), GMMA-OpcA (**b**), rPorB (**c**) and GMMA-NspA (**d**) sera used for Fig. 4 are reported.

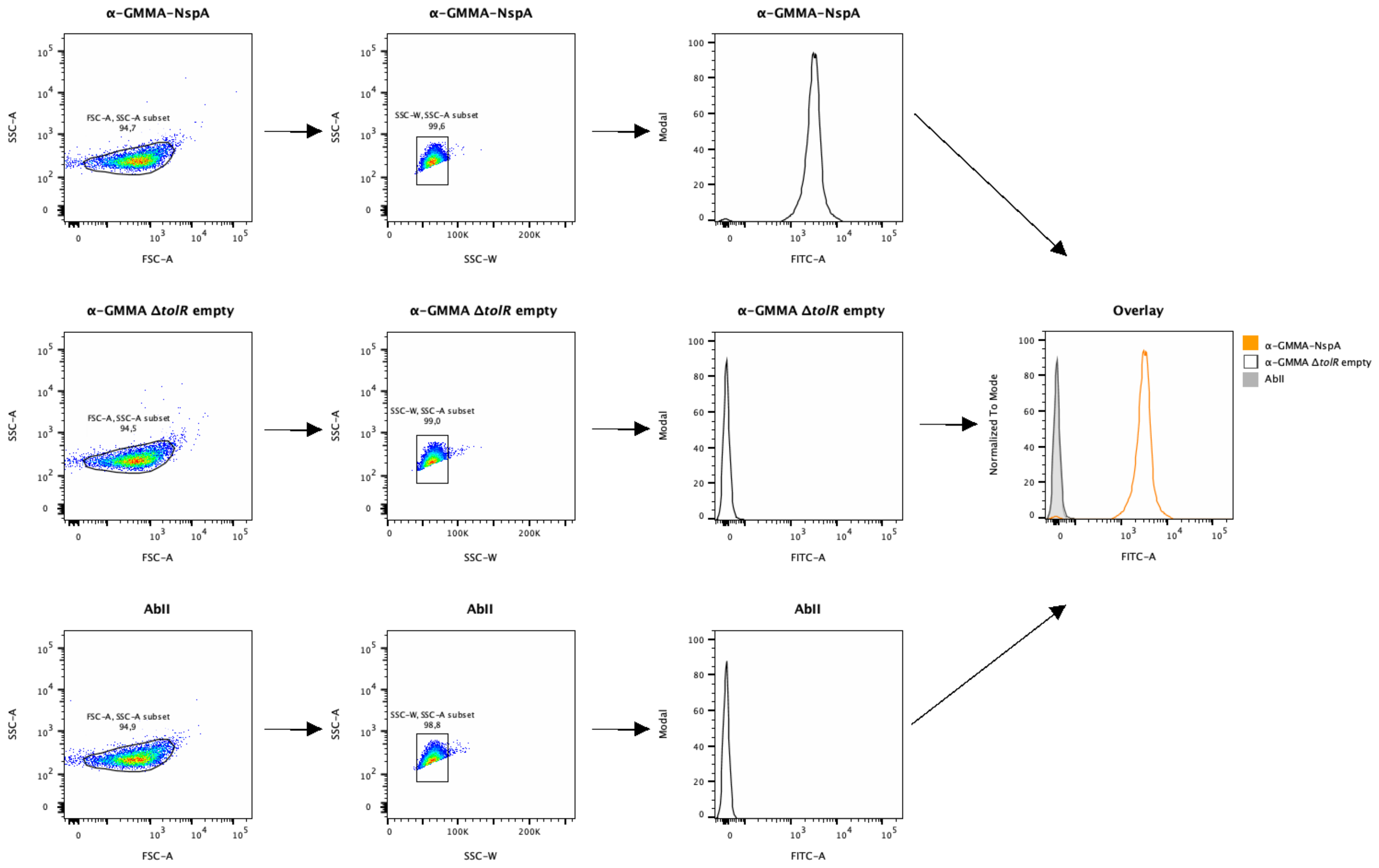

Supplementary Figure 10. Exemplification of the gating strategy used for FACS analyses of Fig.4 and Supplementary Figure 8. The dot plots show the gating strategy used for MenB surface staining, using M07576 as example. Morphology was determined with the FSC-A vs SSC-A, singlets with SSC-W vs SSC-A, the population of interest as FITC$^+$ vs SSC-A, represented as histogram. Finally, the images were represented as the overlay of negative controls (AbII and α-GMMA empty) vs tested sample.

Sera not reactive in protein array with OMV sera	rSBA on three MenB strains		
	NZ98/254	M07576	M09929
GMMA empty Δ*tolR*	<16	<16	<16
GMMA empty Δ*ompA*	<16	<16	<16
GMMA-NEIS0944	<16	<16	<16
GMMA-NEIS0408 (PilQ)	<16	<16	<16
GMMA-NEIS0073 (OmpP1)	<16	<16	<16
GMMA-NEIS1468 (LbpA)	<16	<16	<16
GMMA-NEIS1487 (MIP)	<16	<16	<16
rNEIS1487 (MIP)	<16	<16	<16
GMMA-NEIS2075 (ACP)	<16	<16	<16
rNEIS2075 (ACP)	<16	<16	<16
GMMA-NEIS1246 (PilW)	<16	<16	<16
rNEIS1246 (PilW)	<16	<16	<16
GMMA-NEIS1462 (OmpH8)	<16	<16	<16
rNEIS1462 (OmpH8)	<16	<16	<16
GMMA-NEIS0172 (OmpH)	<16	<16	<16
GMMA-NEIS1066 (Hp)	<16	<16	<16
rNEIS1066 (Hp)	<16	<16	<16
GMMA-NEIS0596 (MafA)	<16	<16	<16
GMMA-NEIS1634 (MtrC)	<16	<16	<16
rNEIS1634 (MtrC)	<16	<16	<16
GMMA-NEIS0653 (ComL)	<16	<16	<16
rNEIS0653 (ComL)	<16	<16	<16
GMMA-NEIS1065 (Hp)	<16	<16	<16
rNEIS1065 (Hp)	<16	<16	<16

Supplementary Table 1. Rabbit SBA screening of polyclonal sera not reactive to OMV according to protein array analysis. NZ98/254, M07576 and M09929 were analysed for their susceptibility to killing by different polyclonal sera through rSBA. Each sample tested is a pool made from 8 individual mouse serum.

Supplementary Table 2. List of strains used in this study.

Name	Description	Antibiotic resistance	Reference
BL21(DE3) Δ*tolR*	BL21(DE3) derivative, lacking *tolR* gene	KanR	[4]
BL21(DE3) Δ*ompA*	BL21(DE3) derivative, lacking *ompA* gene	CmR	[3]
BL21(DE3) Δ*tolR* Empty	BL21(DE3)Δ*tolR* derivative, carryng the pET15b plasmid	KanR	This study
BL21(DE3) Δ*ompA* Empty	BL21(DE3)Δ*ompA* derivative, carryng the pET15b plasmid	CmR	This study
BL21(DE3) Δ*ompA* NEIS2020	BL21(DE3)Δ*ompA* derivative, carryng the pET21-LPompA NEIS2020_N plasmid for the expression of NEIS2020	CmR, AmpR	This study
BL21(DE3) Δ*ompA* NEIS1364	BL21(DE3)Δ*ompA* derivative, carryng the pET21-LPompA NEIS1364_N plasmid for the expression of NEIS1364	CmR, AmpR	This study
BL21(DE3) Δ*tolR* NEIS1428	BL21(DE3)Δ*tolR* derivative, carryng the pET21-LPompA NEIS1428_N plasmid for the expression of NEIS1428	KanR, AmpR	This study
BL21(DE3) Δ*tolR* NEIS1783	BL21(DE3)Δ*tolR* derivative, carryng the pET21-LPompA NEIS1783_C plasmid for the expression of NEIS1783	KanR, AmpR	This study
BL21(DE3) Δ*tolR* NEIS0944	BL21(DE3)Δ*tolR* derivative, carryng the pET21-LPompA NEIS0944_N plasmid for the expression of NEIS0944	KanR, AmpR	This study
BL21(DE3) Δ*tolR* NEIS0408	BL21(DE3)Δ*tolR* derivative, carryng the pET21-LPompA NEIS0408_C plasmid for the expression of NEIS0408	KanR, AmpR	This study
BL21(DE3) Δ*tolR* NEIS2198	BL21(DE3)Δ*tolR* derivative, carryng the pET21-LPompA NEIS2198_N plasmid for the expression of NEIS2198	KanR, AmpR	This study
BL21(DE3) Δ*tolR* NEIS1963	BL21(DE3)Δ*tolR* derivative, carryng thepET21-LPompA NEIS1963_N plasmid for the expression of NEIS1963	KanR, AmpR	This study
BL21(DE3) Δ*tolR* NEIS1690_C	BL21(DE3)Δ*tolR* derivative, carryng the pET21-LPompA NEIS1690_C plasmid for the expression of NEIS1690	KanR, AmpR	This study
BL21(DE3) Δ*tolR* NEIS1690_N	BL21(DE3)Δ*tolR* derivative, carryng the pET21-LPompA NEIS1690_N plasmid for the expression of NEIS1690	KanR, AmpR	This study
BL21(DE3) Δ*tolR* NEIS0173	BL21(DE3)Δ*tolR* derivative, carryng the pET21-LPompA NEIS0173_N plasmid for the expression of NEIS0173	KanR, AmpR	This study
BL21(DE3) Δ*tolR* NEIS0073	BL21(DE3)Δ*tolR* derivative, carryng the pET21-LPompA NEIS0073_N plasmid for the expression of NEIS0073	KanR, AmpR	This study
BL21(DE3) Δ*tolR* NEIS1468	BL21(DE3)Δ*tolR* derivative, carryng the pET21-LPompA NEIS1468_N plasmid for the expression of NEIS1468	KanR, AmpR	This study
BL21(DE3) Δ*tolR* NEIS0275_C	BL21(DE3)Δ*tolR* derivative, carryng the pET21-LPompA NEIS0275_C plasmid for the expression of NEIS0275	KanR, AmpR	This study
BL21(DE3) Δ*tolR* NEIS0275_N	BL21(DE3)Δ*tolR* derivative, carryng the pET21-LPompA NEIS0275_N plasmid for the expression of NEIS0275	KanR, AmpR	This study
BL21(DE3) Δ*tolR* NEIS1487	BL21(DE3)Δ*tolR* derivative, carryng the pET21-LPompA NEIS1487_C plasmid for the expression of NEIS1487	KanR, AmpR	This study
BL21(DE3) Δ*tolR* NEIS1632	BL21(DE3)Δ*tolR* derivative, carryng the pET21-LPompA NEIS1632_C plasmid for the expression of NEIS1632	KanR, AmpR	This study
BL21(DE3) Δ*tolR* NEIS0101_C	BL21(DE3)Δ*tolR* derivative, carryng the pET21-LPompA NEIS0101_C plasmid for the expression of NEIS0101	KanR, AmpR	This study

BL21(DE3) Δ*tolR* NEIS0101_N	BL21(DE3)Δ*tolR* derivative, carryng the pET21-LPompA NEIS0101_N plasmid for the expression of NEIS0101	KanR, AmpR	This study
BL21(DE3) Δ*tolR* NEIS1271	BL21(DE3)Δ*tolR* derivative, carryng the pET21-LPompA NEIS1271_C plasmid for the expression of NEIS1271	KanR, AmpR	This study
BL21(DE3) Δ*tolR* NEIS1687	BL21(DE3)Δ*tolR* derivative, carryng the pET21-LPompA NEIS1687_N plasmid for the expression of NEIS1687	KanR, AmpR	This study
BL21(DE3) Δ*tolR* NEIS0210_C	BL21(DE3)Δ*tolR* derivative, carryng the pET21-LPompA NEIS0210_C plasmid for the expression of NEIS0210	KanR, AmpR	This study
BL21(DE3) Δ*tolR* NEIS0210_N	BL21(DE3)Δ*tolR* derivative, carryng the pET21-LPompA NEIS0210_N plasmid for the expression of NEIS0210	KanR, AmpR	This study
BL21(DE3) Δ*tolR* NEIS2075	BL21(DE3)Δ*tolR* derivative, carryng the pET21-LPompA NEIS2075_C plasmid for the expression of NEIS2075	KanR, AmpR	This study
BL21(DE3) Δ*tolR* NEIS1246	BL21(DE3)Δ*tolR* derivative, carryng the pET21-LPompA NEIS1246_C plasmid for the expression of NEIS1246	KanR, AmpR	This study
BL21(DE3) Δ*tolR* NEIS0612	BL21(DE3)Δ*tolR* derivative, carryng the pET21-LPompA NEIS0612_N plasmid for the expression of NEIS0612	KanR, AmpR	This study
BL21(DE3) Δ*tolR* NEIS1462	BL21(DE3)Δ*tolR* derivative, carryng the pET21-LPompA NEIS1462_C plasmid for the expression of NEIS1462	KanR, AmpR	This study
BL21(DE3) Δ*tolR* NEIS0172	BL21(DE3)Δ*tolR* derivative, carryng the pET21-LPompA NEIS0172_C plasmid for the expression of NEIS0172	KanR, AmpR	This study
BL21(DE3) Δ*tolR* NEIS1066	BL21(DE3)Δ*tolR* derivative, carryng the pET21-LPompA NEIS1066_C plasmid for the expression of NEIS1066	KanR, AmpR	This study
BL21(DE3) Δ*tolR* NEIS0596	BL21(DE3)Δ*tolR* derivative, carryng the pET21-LPompA NEIS0596_N plasmid for the expression of NEIS0596	KanR, AmpR	This study
BL21(DE3) Δ*tolR* NEIS1634	BL21(DE3)Δ*tolR* derivative, carryng the pET21-LPompA NEIS1634_C plasmid for the expression of NEIS1634	KanR, AmpR	This study
BL21(DE3) Δ*tolR* NEIS0653	BL21(DE3)Δ*tolR* derivative, carryng the pET21-LPompA NEIS0653_C plasmid for the expression of NEIS0653	KanR, AmpR	This study
BL21(DE3) Δ*tolR* NEIS0196	BL21(DE3)Δ*tolR* derivative, carryng the pET21-LPompA NEIS0196_C plasmid for the expression of NEIS0196	KanR, AmpR	This study
BL21(DE3) Δ*tolR* NEIS1065	BL21(DE3)Δ*tolR* derivative, carryng the pET21-LPompA NEIS1065_C plasmid for the expression of NEIS1065	KanR, AmpR	This study
NZ98/254	Clinical isolate	/	/
M08389	Clinical isolate	/	/
LNP24651	Clinical isolate	/	/
M08117	Clinical isolate	/	/
M10837	Clinical isolate	/	/
MC58	*Neisseria meningitidis* laboratory-adapted reference strain	/	[1]
M09662	Clinical isolate	/	/
M14569	Clinical isolate	/	/
M07576	Clinical isolate	/	/
M09929	Clinical isolate	/	/
M12898	Clinical isolate	/	/
M07 241084	Clinical isolate	/	/
NZ98/254 Δ*porA*	NZ98/254 derivative, lacking *porA* gene	SpecR	This study
LNP24651 Δ*opcA*	LNP24651 derivative, lacking *opcA* gene	KanR	This study
M07576 Δ*nspA*	M07576 derivative, lacking *nspA* gene	EryR	This study

AmpR, ampicillin resistance; CmR, chloramphenicol resistance; EryR, erythromycin resistance; KanR, kanamycin resistance; SpecR, spectinomycin resistance.

Supplementary Table 3. Meningococcal antigens cloned in this study.

PubMLST	Leader sequence		Mw (kDa)	Tag (6xHis + FLAG)	*E. coli* cells used for transformation
	Removed	Amino acid residues removed			
NEIS2020	yes	1-19	35.7	N-term	BL21(DE3) Δ*ompA*
NEIS1364	yes	1-19	41.6	N-term	BL21(DE3) Δ*ompA*
NEIS1428	yes	1-25	103	N-term	BL21(DE3) Δ*tolR*
NEIS1783	yes	1-22	25.7	C-term	BL21(DE3) Δ*tolR*
NEIS0944	yes	1-24	84.3	N-term	BL21(DE3) Δ*tolR*
NEIS0408	yes	1-24	82	C-term	BL21(DE3) Δ*tolR*
NEIS2198	yes	1-19	30	N-term	BL21(DE3) Δ*tolR*
NEIS1963	yes	1-22	78.7	N-term	BL21(DE3) Δ*tolR*
NEIS1690	yes	1-24	101.3	C-term/N-term	BL21(DE3) Δ*tolR*
NEIS0173	yes	1-21	88.2	N-term	BL21(DE3) Δ*tolR*
NEIS0073	yes	1-24	50.2	N-term	BL21(DE3) Δ*tolR*
NEIS1468	yes	1-24	105.2	N-term	BL21(DE3) Δ*tolR*
NEIS0275	yes	1-23	88	C-term/N-term	BL21(DE3) Δ*tolR*
NEIS1487	yes	1-22	28.7	C-term	BL21(DE3) Δ*tolR*
NEIS1632	yes	1-39	50.2	C-term	BL21(DE3) Δ*tolR*
NEIS0101	no	-	52	C-term/N-term	BL21(DE3) Δ*tolR*
NEIS1271	yes	1-20	66.2	C-term	BL21(DE3) Δ*tolR*
NEIS1687	yes	1-22	41.5	N-term	BL21(DE3) Δ*tolR*
NEIS0210	no	-	19.4	C-term/N-term	BL21(DE3) Δ*tolR*
NEIS2075	yes	1-21	13.2	C-term	BL21(DE3) Δ*tolR*
NEIS1246	yes	1-37	27.6	C-term	BL21(DE3) Δ*tolR*
NEIS0612	yes	1-19	18.5	N-term	BL21(DE3) Δ*tolR*
NEIS1462	yes	1-23	18.2	C-term	BL21(DE3) Δ*tolR*
NEIS0172	yes	1-23	18.8	C-term	BL21(DE3) Δ*tolR*
NEIS1066	yes	1-22	23.8	C-term	BL21(DE3) Δ*tolR*
NEIS0596	yes	1-25	33.2	N-term	BL21(DE3) Δ*tolR*
NEIS1634	yes	1-26	42.3	C-term	BL21(DE3) Δ*tolR*
NEIS0653	yes	1-18	31	C-term	BL21(DE3) Δ*tolR*
NEIS0196	yes	1-18	14.01	C-term	BL21(DE3) Δ*tolR*
NEIS1065	yes	1-24	13.1	C-term	BL21(DE3) Δ*tolR*

Supplementary Table 4. List of primers used in this study.

Name	Sequence	Application	Reference
HISFLAG Fw	catcaccatcaccatCACGATTACAAAGAC	vPCR for cloning the antigens with a C-terminal Tag (Fw)	This study
OmpRev	ggcctgcgctacggtAGCGAAA	vPCR for cloning the antigens with a C-terminal Tag (Rev)	This study
For-pet21	tgagatccggctgcTAACAAAGCCCGAAAGG	vPCR for cloning the antigens with a N-terminal Tag (Fw)	This study
HisFlagRev-pet21	cttgtcatcatcgtcTTTGTAATCGTGATGGTGATGG	vPCR for cloning the antigens with a N-terminal Tag (Rev)	This study
NEIS2020-Fw_n	gacgatgatgacaagGACGTTACCCTGTACGGCACCA	iPCR for cloning of NEIS2020 with the N-terminal Tag (Fw)	This study
NEIS2020-Rv_n	gcagccggatctcaGAATTTGTGGCGCAGACCGACA	iPCR for cloning of NEIS2020 with the N-terminal Tag (Rev)	This study
NEIS1364-Fw_n	gacgatgatgacaagGATGTCAGCCTATACGGCGAAAT	iPCR for cloning of NEIS1364 with the N-terminal Tag (Fw)	This study
NEIS1364-Rv_n	gcagccggatctcaGAATTTGTGGCGCAAACCGACG	iPCR for cloning of NEIS1364 with the N-terminal Tag (Rev)	This study
NEIS1428-Fw_n	gacgatgatgacaagGAAGATGCAGGGCGCGCGGG	iPCR for cloning of NEIS1428 with the N-terminal Tag (Fw)	This study
NEIS1428-Rv_n	gcagccggatctcaAAACTTGTAGCTCATCGTTATCAAAA	iPCR for cloning of NEIS1428 with the N-terminal Tag (Rev)	This study
NEIS1783-Fw_c	accgtagcgcaggccGGCGAGGCGTCCGTTCAG	iPCR for cloning of NEIS1783 with the C-terminal Tag (Fw)	This study
NEIS1783-Rv_c	atggtgatggtgatgGTGTTGGTGATGATTGTG	iPCR for cloning of NEIS1783 with the C-terminal Tag (Rev)	This study
NEIS0944-Fw_n	gacgatgatgacaagCATGAAACCGAGCAGTCGGTGG	iPCR for cloning of NEIS0944 with the N-terminal Tag (Fw)	This study
NEIS0944-Rv_n	gcagccggatctcaAAACTTCACGTTCACGCCGCCG	iPCR for cloning of NEIS0944 with the N-terminal Tag (Rev)	This study
NEIS0408-Fw_c	accgtagcgcaggccGGAAACATTACAGACATCAAAGTTTC	iPCR for cloning of NEIS0408 with the C-terminal Tag (Fw)	This study
NEIS0408-Rv_c	atggtgatggtgatgATAGCGCAGGCTGTTGCCGGC	iPCR for cloning of NEIS0408 with the C-terminal Tag (Rev)	This study
NEIS2198-Fw_n	gacgatgatgacaagGCA CAAGAGCTTC AAACCGC	iPCR for cloning of NEIS2198 with the N-terminal Tag (Fw)	This study
NEIS2198-Rv_n	gcagccggatctca GAATTTTATGCCGACGCGCAAG	iPCR for cloning of NEIS2198 with the N-terminal Tag (Rev)	This study
NEIS1963-Fw_n	gacgatgatgacaagGCAGAAAATAATGCCAAGGTCGTA	iPCR for cloning of NEIS1963 with the N-terminal Tag (Fw)	This study
NEIS1963-Rv_n	gcagccggatctcaGAACTTGTAGTTCACGCCCAAGC	iPCR for cloning of NEIS1963 with the N-terminal Tag (Rev)	This study
NEIS1690-Fw_c	accgtagcgcaggccGAAAATGTGCAAGCCGGACAAG	iPCR for cloning of NEIS1690 with the C-terminal Tag (Fw)	This study
NEIS1690-Rv_c	atggtgatggtgatgGAACTTCATTTCCAAGCTAAATG	iPCR for cloning of NEIS1690 with the C-terminal Tag (Rev)	This study
NEIS1690-Fw_n	gacgatgatgacaagGAAAATGTGCAAGCCGGACAAG	iPCR for cloning of NEIS1690 with the N-terminal Tag (Fw)	This study
NEIS1690-Rv_n	gcagccggatctcaGAACTTCATTTCCAAGCTAAATG	iPCR for cloning of NEIS1690 with the N-terminal Tag (Rev)	This study
NEIS0173-Fw_n	gacgatgatgacaagGACTTCACCATCCAAGACATCCG	iPCR for cloning of NEIS0173 with the N-terminal Tag (Fw)	This study
NEIS0173-Rv_n	gcagccggatctcaGAACGTCGTGCCGAGTTGGAATT	iPCR for cloning of NEIS0173 with the N-terminal Tag (Rev)	This study
NEIS0073-Fw_n	gacgatgatgacaagTCCGGCTACCACTTCGGCACA	iPCR for cloning of NEIS0073 with the N-terminal Tag (Fw)	This study
NEIS0073-Rv_n	gcagccggatctcaTTTGAATTTGTAGGTGTATTGCAGG	iPCR for cloning of NEIS0073 with the N-terminal Tag (Rev)	This study
NEIS1468-Fw_n	gacgatgatgacaagGCAAACCCTGAAACGGCGGCA	iPCR for cloning of NEIS1468 with the N-terminal Tag (Fw)	This study
NEIS1468-Rv_n	gcagccggatctcaAAACTTCATTTCGAGCGCGAGGC	iPCR for cloning of NEIS1468 with the N-terminal Tag (Rev)	This study
NEIS0275-Fw_c	accgtagcgcaggccGCCGCCGATGCCGTTGCG	iPCR for cloning of NEIS0275 with the C-terminal Tag (Fw)	This study

NEIS0275-Rv_c	atggtgatggtgatgGGGTCGTTTGTTGCGTCCGG	iPCR for cloning of NEIS0275 with the C-terminal Tag (Rev)	This study
NEIS0275-Fw_n	gacgatgatgacaagGCCGCCGATGCCGTTGCG	iPCR for cloning of NEIS0275 with the N-terminal Tag (Fw)	This study
NEIS0275-Rv_n	gcagccggatctcaGGGTCGTTTGTTGCGTCCGG	iPCR for cloning of NEIS0275 with the N-terminal Tag (Rev)	This study
NEIS1487-Fw_c	accgtagcgcaggccAAAAAAGAAGCCGCCCCCGCAT	iPCR for cloning of NEIS1487 with the C-terminal Tag (Fw)	This study
NEIS1487-Rv_c	atggtgatggtgatgATTTACTTTTTTGATGTCGACTTGAG	iPCR for cloning of NEIS1487 with the C-terminal Tag (Rev)	This study
NEIS1632-Fw_c	accgtagcgcaggccATTCCCCAATACGAGCAGC	iPCR for cloning of NEIS1632 with the C-terminal Tag (Fw)	This study
NEIS1632-Rv_c	atggtgatggtgatgTTTGTCGGTTTGGGTATCC	iPCR for cloning of NEIS1632 with the C-terminal Tag (Rev)	This study
NEIS0101-Fw_c	accgtagcgcaggccTTAAAATGCGGAACATTTTTTATC	iPCR for cloning of NEIS0101 with the C-terminal Tag (Fw)	This study
NEIS0101-Rv_c	atggtgatggtgatgTTCGGAGCGGTTGAAGCCAAAC	iPCR for cloning of NEIS0101 with the C-terminal Tag (Rev)	This study
NEIS0101-Fw_n	gacgatgatgacaagTTAAAATGCGGAACATTTTTTATC	iPCR for cloning of NEIS0101 with the N-terminal Tag (Fw)	This study
NEIS0101-Rv_n	gcagccggatctcaTTCGGAGCGGTTGAAGCCAAAC	iPCR for cloning of NEIS0101 with the N-terminal Tag (Rev)	This study
NEIS1271-Fw_c	accgtagcgcaggccGCCCACGACGCGGCACACAA	iPCR for cloning of NEIS1271 with the C-terminal Tag (Fw)	This study
NEIS1271-Rv_c	atggtgatggtgatg ACGTATCCAGCTCGAAGGGTTCA	iPCR for cloning of NEIS1271 with the C-terminal Tag (Rev)	This study
NEIS1687-Fw_n	gacgatgatgacaagGAGACCGCGCTGCAATGCGC	iPCR for cloning of NEIS1687 with the N-terminal Tag (Fw)	This study
NEIS1687-Rv_n	gcagccggatctcaGATGCCGTCCAAGTCGTTGAACA	iPCR for cloning of NEIS1687 with the N-terminal Tag (Rev)	This study
NEIS0210-Fw_c	accgtagcgcaggccAACACCCTTCAAAAAGGTTTTAC	iPCR for cloning of NEIS0210 with the C-terminal Tag (Fw)	This study
NEIS0210-Rv_c	atggtgatggtgatgGCTGGCAGATGCTGTGTCG	iPCR for cloning of NEIS0210 with the C-terminal Tag (Rev)	This study
NEIS0210-Fw_n	gacgatgatgacaagAACACCCTTCAAAAAGGTTTTAC	iPCR for cloning of NEIS0210 with the N-terminal Tag (Fw)	This study
NEIS0210-Rv_n	gcagccggatctcaGCTGGCAGATGCTGTGTCG	iPCR for cloning of NEIS0210 with the N-terminal Tag (Rev)	This study
NEIS2075-Fw_c	accgtagcgcaggccGCCGGCACGAACAACCCCAC	iPCR for cloning of NEIS2075 with the C-terminal Tag (Fw)	This study
NEIS2075-Rv_c	atggtgatggtgatg ACGTGGGGAACAGTCTTTGAAGA	iPCR for cloning of NEIS2075 with the C-terminal Tag (Rev)	This study
NEIS1246-Fw_c	accgtagcgcaggccCCCTCGCGGGCAGAAAAAGCC	iPCR for cloning of NEIS1246 with the C-terminal Tag (Fw)	This study
NEIS1246-Rv_c	atggtgatggtgatgTTGACCGGTGAGGACGGTTTGC	iPCR for cloning of NEIS1246 with the C-terminal Tag (Rev)	This study
NEIS0612-Fw_n	gacgatgatgacaagGAAGGCGCATCCGGCTTTTACG	iPCR for cloning of NEIS0612 with the N-terminal Tag (Fw)	This study
NEIS0612-Rv_n	gcagccggatctcaGAATTTGACGCGCACACCGGC	iPCR for cloning of NEIS0612 with the N-terminal Tag (Rev)	This study
NEIS1462-Fw_c	accgtagcgcaggccGCGCCTGCTGCCGAGGCAAC	iPCR for cloning of NEIS1462 with the C-terminal Tag (Fw)	This study
NEIS1462-Rv_c	atggtgatggtgatgATCGACCAAAGTCACTTTGCCGTT	iPCR for cloning of NEIS1462 with the C-terminal Tag (Rev)	This study
NEIS0172-Fw_c	accgtagcgcaggccGACACCTTCCAAAAAAATCGGCTTT	iPCR for cloning of NEIS0172 with the C-terminal Tag (Fw)	This study
NEIS0172-Rv_c	atggtgatggtgatgGCGGGCGTTCATTTCTTTAATGAC	iPCR for cloning of NEIS0172 with the C-terminal Tag (Rev)	This study
NEIS1066-Fw_c	accgtagcgcaggccGAATCCTCACGCAGTCTCG	iPCR for cloning of NEIS1066 with the C-terminal Tag (Fw)	This study
NEIS1066-Rv_c	atggtgatggtgatgACGGTTGGGTTGCCATG	iPCR for cloning of NEIS1066 with the C-terminal Tag (Rev)	This study
NEIS0596-Fw_n	gacgatgatgacaagGGCGGCAAACGCTTTGCCG	iPCR for cloning of NEIS0596 with the N-terminal Tag (Fw)	This study
NEIS0596-Rv_n	gcagccggatctcaTCCTCCTTTGCGGCGGCGG	iPCR for cloning of NEIS0596 with the N-terminal Tag (Rev)	This study
NEIS1634-Fw_c	accgtagcgcaggccAAAGGCGGAGACGCGGCGC	iPCR for cloning of NEIS1634 with the C-terminal Tag (Fw)	This study
NEIS1634-Rv_c	atggtgatggtgatgTTCCGCTTCAGAAGCAGTTTTGG	iPCR for cloning of NEIS1634 with the C-terminal Tag (Rev)	This study

NEIS0653-Fw_c	accgtagcgcaggccACTCAAGGTACGGTCGATAAAGATG	iPCR for cloning of NEIS0653 with the C-terminal Tag (Fw)	This study
NEIS0653-Rv_c	atggtgatggtgatgATGCCAGTAACGCCACCAGGGC	iPCR for cloning of NEIS0653 with the C-terminal Tag (Rev)	This study
NEIS0196-Fw_c	accgtagcgcaggccAGCGTCGAACGCGTTTCAC	iPCR for cloning of NEIS0196 with the C-terminal Tag (Fw)	This study
NEIS0196-Rv_c	atggtgatggtgatgGAATTTTATGCCGACGCG	iPCR for cloning of NEIS0196 with the C-terminal Tag (Rev)	This study
NEIS1065-Fw_c	accgtagcgcaggccAACGGGCA AAAATCCCTG TA	iPCR for cloning of NEIS1065 with the C-terminal Tag (Fw)	This study
NEIS1065-Rv_c	atggtgatggtgatgTCGCTTGCCTCCTTTAC	iPCR for cloning of NEIS1065 with the C-terminal Tag (Rev)	This study
porAkoUPF	ctgtacttccagggcGAATCTAGGACGCAGGGTTAAG	Amplification of *porA* promoter and CDS (Fw)	This study
porAkoDOR	aattaagtcgcgttaAAGTCCGGAGAGTCGTAGCGTAC	Amplification of *porA* promoter and CDS (Rev)	This study
porAkoUPR	gctgctgcccggatgCAGTCTC	vPCR for *porA* knock-out mutant (Rev)	This study
porAkoDOF	tattactagtcgacaACTTACCGCCCTCGTATTGTC	vPCR for *porA* knock-out mutant (Fw)	This study
porAkoDOR	aattaagtcgcgttaAAGTCCGGAGAGTCGTAGCGTAC	Amplification of porA promoter and CDS (Rev)	This study
CSporAF	catccgggcagcagcGACATCTTGGTTACCGTGAAG	iPCR of *tetR-sacB-specR* selection cassette (Fw)	This study
CSporAR	cggtattgcgtttcaTTCGCGGTGGCTGAGATCAG	iPCR of *tetR-sacB-specR* selection cassette (Rev)	This study

Capital letters correspond to nucleotides of the meningococcal sequence or of the vectors, whereas small letters of each primer sequence correspond to the 15 base complementary overlaps used for PIPE-cloning.

Supplementary Table 5. List of plasmids used in this study.

Name	Description	Antibiotic resistance	Reference
pET15-LPompA 6xHis+FLAG	pET15 derivative containing the LP of *E. coli* ompA followed by 6 Histidine residues and the FLAG tag	AmpR	This study
pET21-LPompA 6xHis+FLAG	pET21 derivative containing the LP of *E. coli* ompA followed by 6 Histidine residues and the FLAG tag	AmpR	This study
pET21-LPompA NEIS2020_N	pET21-LPompA 6xHis+FLAG. Costruct for NEIS2020 expression with N-terminal tag	AmpR	This study
pET21-LPompA NEIS1364_N	pET21-LPompA 6xHis+FLAG. Costruct for NEIS1364 expression with N-terminal tag	AmpR	This study
pET21-LPompA NEIS1428_N	pET21-LPompA 6xHis+FLAG. Costruct for NEIS1428 expression with N-terminal tag	AmpR	This study
pET21-LPompA NEIS1783_C	pET21-LPompA 6xHis+FLAG. Costruct for NEIS1783 expression with C-terminal tag	AmpR	This study
pET21-LPompA NEIS0944_N	pET21-LPompA 6xHis+FLAG. Costruct for NEIS0944 expression with N-terminal tag	AmpR	This study
pET21-LPompA NEIS0408_C	pET21-LPompA 6xHis+FLAG. Costruct for NEIS0408 expression with C-terminal tag	AmpR	This study
pET21-LPompA NEIS2198_N	pET21-LPompA 6xHis+FLAG. Costruct for NEIS2198 expression with N-terminal tag	AmpR	This study
pET21-LPompA NEIS1963_N	pET21-LPompA 6xHis+FLAG. Costruct for NEIS1963 expression with N-terminal tag	AmpR	This study
pET21-LPompA NEIS1690_C	pET21-LPompA 6xHis+FLAG. Costruct for NEIS1690 expression with C-terminal tag	AmpR	This study
pET21-LPompA NEIS1690_N	pET21-LPompA 6xHis+FLAG. Costruct for NEIS1690 expression with N-terminal tag	AmpR	This study
pET21-LPompA NEIS0173_N	pET21-LPompA 6xHis+FLAG. Costruct for NEIS0173 expression with N-terminal tag	AmpR	This study
pET21-LPompA NEIS0073_N	pET21-LPompA 6xHis+FLAG. Costruct for NEIS0073 expression with N-terminal tag	AmpR	This study
pET21-LPompA NEIS1468_N	pET21-LPompA 6xHis+FLAG. Costruct for NEIS1468 expression with N-terminal tag	AmpR	This study
pET21-LPompA NEIS0275_C	pET21-LPompA 6xHis+FLAG. Costruct for NEIS0275 expression with C-terminal tag	AmpR	This study
pET21-LPompA NEIS0275_N	pET21-LPompA 6xHis+FLAG. Costruct for NEIS0275 expression with N-terminal tag	AmpR	This study
pET21-LPompA NEIS1487_C	pET21-LPompA 6xHis+FLAG. Costruct for NEIS1487 expression with C-terminal tag	AmpR	This study
pET21-LPompA NEIS1632_C	pET21-LPompA 6xHis+FLAG. Costruct for NEIS1632 expression with C-terminal tag	AmpR	This study
pET21-LPompA NEIS0101_C	pET21-LPompA 6xHis+FLAG. Costruct for NEIS0101 expression with C-terminal tag	AmpR	This study
pET21-LPompA NEIS0101_N	pET21-LPompA 6xHis+FLAG. Costruct for NEIS0101 expression with N-terminal tag	AmpR	This study
pET21-LPompA NEIS1271_C	pET21-LPompA 6xHis+FLAG. Costruct for NEIS1271 expression with C-terminal tag	AmpR	This study
pET21-LPompA NEIS1687_N	pET21-LPompA 6xHis+FLAG. Costruct for NEIS1687 expression with N-terminal tag	AmpR	This study
pET21-LPompA NEIS0210_C	pET21-LPompA 6xHis+FLAG. Costruct for NEIS0210 expression with C-terminal tag	AmpR	This study
pET21-LPompA NEIS0210_N	pET21-LPompA 6xHis+FLAG. Costruct for NEIS0210 expression with N-terminal tag	AmpR	This study
pET21-LPompA NEIS2075_C	pET21-LPompA 6xHis+FLAG. Costruct for NEIS2075 expression with C-terminal tag	AmpR	This study
pET21-LPompA NEIS1246_C	pET21-LPompA 6xHis+FLAG. Costruct for NEIS1246 expression with C-terminal tag	AmpR	This study
pET21-LPompA NEIS0612_N	pET21-LPompA 6xHis+FLAG. Costruct for NEIS0612 expression with N-terminal tag	AmpR	This study
pET21-LPompA NEIS1462_C	pET21-LPompA 6xHis+FLAG. Costruct for NEIS1462 expression with C-terminal tag	AmpR	This study

pET21-LPompA NEIS0172_C	pET21-LPompA 6xHis+FLAG. Costruct for NEIS0172 expression with C-terminal tag	AmpR	This study
pET21-LPompA NEIS1066_C	pET21-LPompA 6xHis+FLAG. Costruct for NEIS1066 expression with C-terminal tag	AmpR	This study
pET21-LPompA NEIS0596_N	pET21-LPompA 6xHis+FLAG. Costruct for NEIS0596 expression with N-terminal tag	AmpR	This study
pET21-LPompA NEIS1634_C	pET21-LPompA 6xHis+FLAG. Costruct for NEIS1634 expression with C-terminal tag	AmpR	This study
pET21-LPompA NEIS0653_C	pET21-LPompA 6xHis+FLAG. Costruct for NEIS0653 expression with C-terminal tag	AmpR	This study
pET21-LPompA NEIS0196_C	pET21-LPompA 6xHis+FLAG. Costruct for NEIS0196 expression with C-terminal tag	AmpR	This study
pET21-LPompA NEIS1065_C	pET21-LPompA 6xHis+FLAG. Costruct for NEIS1065 expression with C-terminal tag	AmpR	This study
pET15-Δ*porA* SpecR	pET15 with the UP and DOWN por*A*-regions for the recombination which contains a Spec resistance cassette	AmpR, SpecR	This study
pBS-Δ*opcA* KanR	pBS with the UP and DOWN *opcA*-regions for the recombination which contains a Kan resistance cassette	AmpR, KanR	[2]
pBS-Δ*nspA* EryR	pBS with the UP and DOWN *nspA*-regions for the recombination which contains a Ery resistance cassette	AmpR, EryR	[2]

AmpR, ampicillin resistance; EryR, erythromycin resistance; KanR, kanamycin resistance; SpecR, spectinomycin resistance.

References

1 Tettelin, H. *et al.* Complete genome sequence of Neisseria meningitidis serogroup B strain MC58. *Science* **287**, 1809-1815, doi:10.1126/science.287.5459.1809 (2000).

2 Echenique-Rivera, H. *et al.* Transcriptome analysis of Neisseria meningitidis in human whole blood and mutagenesis studies identify virulence factors involved in blood survival. *PLoS Pathog* **7**, e1002027, doi:10.1371/journal.ppat.1002027 (2011).

3 Fantappie, L. *et al.* Antibody-mediated immunity induced by engineered Escherichia coli OMVs carrying heterologous antigens in their lumen. *J Extracell Vesicles* **3**, doi:10.3402/jev.v3.24015 (2014).

4 Berlanda Scorza, F. *et al.* Proteomics characterization of outer membrane vesicles from the extraintestinal pathogenic Escherichia coli DeltatolR IHE3034 mutant. *Mol Cell Proteomics* **7**, 473-485, doi:10.1074/mcp.M700295-MCP200 (2008).
